# The transcriptional regulator KLF15 is necessary for myoblast differentiation and muscle regeneration by activating FKBP5

**DOI:** 10.1016/j.jbc.2023.105226

**Published:** 2023-09-04

**Authors:** Shijuan Gao, Shan Huang, Yanhong Zhang, Guangming Fang, Yan Liu, Congcong Zhang, Yulin Li, Jie Du

**Affiliations:** Collaborative Innovation Centre for Cardiovascular Disorders, The Key Laboratory of Remodeling-Related Cardiovascular Diseases, Ministry of Education, Beijing Institute of Heart, Lung and Blood Vessel Diseases, Beijing Anzhen Hospital, Capital Medical University, Beijing, China

**Keywords:** muscle regeneration, myoblast differentiation, KLF15, FKBP5, myotube maturation

## Abstract

Successful muscle regeneration following injury is essential for functional homeostasis of skeletal muscles. Krüppel-like factor 15 (KLF15) is a metabolic transcriptional regulator in the muscles. However, little is known regarding its function in muscle regeneration. Here, we examined microarray datasets from the Gene Expression Omnibus database, which indicated downregulated KLF15 in muscles from patients with various muscle diseases. Additionally, we found that *Klf15* knockout (Klf15KO) impaired muscle regeneration following injury in mice. Furthermore, KLF15 expression was robustly induced during myoblast differentiation. Myoblasts with KLF15 deficiency showed a marked reduction in their fusion capacity. Unbiased transcriptome analysis of muscles on day 7 postinjury revealed downregulated genes involved in cell differentiation and metabolic processes in Klf15KO muscles. The FK506-binding protein 51 (FKBP5), a positive regulator of myoblast differentiation, was ranked as one of the most strongly downregulated genes in the Klf15KO group. A mechanistic search revealed that KLF15 binds directly to the promoter region of FKBP5 and activates FKBP5 expression. Local delivery of FKBP5 rescued the impaired muscle regeneration in Klf15KO mice. Our findings reveal a positive regulatory role of KLF15 in myoblast differentiation and muscle regeneration by activating FKBP5 expression. KLF15 signaling may be a novel therapeutic target for muscle disorders associated with injuries or diseases.

The skeletal muscle is the most abundant body tissue and performs critical functions in the human body. To maintain the body’s integrity and homeostasis, skeletal muscles show robust regenerative capacity in response to injury, disease, and aging (1, 2, 3). Regeneration of skeletal muscle is mediated by a specialized population of stem/progenitor cells termed satellite cells (4, 5). Upon injury, quiescent satellite cells become mitotically active and proliferate (termed myoblasts), then withdraw from the cell cycle to begin differentiation, and finally fuse together to form multinucleated myofibers (2). Abnormalities in any stage of regeneration can delay the recovery from muscle injury, aggravate muscle diseases, or contribute to the global burden of metabolic diseases, such as obesity and diabetes (6, 7).

The Krüppel-like factor (KLF) family consists of zinc finger transcription factors with essential functions in a multitude of cellular growth, development, and differentiation. Recently, several members of the KLF family have been suggested to be involved in skeletal muscle regeneration. For instance, KLF5 promotes myoblast differentiation by acting in coordination with MyoD (8). KLF10 inhibits myoblast proliferation by interacting with the FGFR1 promoter and repressing its activity (9). For KLF15, its function in muscle has emerged as a key transcriptional regulator of a gene program governing skeletal muscle metabolism (10). KLF15 has also been described as a positive regulator of nephric tubule regeneration through activating regeneration-specific open chromatin elements (11, 12). Whether KLF15 functions during myoblast differentiation and muscle regeneration remains unclear.

To identify the candidate genes involved in skeletal muscle injury and diseases, we resorted to Gene Expression Omnibus database of muscular diseases (13, 14) to screen differently expressed genes. The downregulated molecule KLF15 was noticed due to its high expression in human skeletal muscle but significant reduction in patients with various muscle diseases including juvenile dermatomyositis (JDM), amyotrophic lateral sclerosis (ALS), Becker muscular dystrophy (BMD), and Duchenne muscular dystrophy (DMD). Considering that regeneration is a unique adaptation of the muscle in response to injury or disease, we hypothesized that KLF15 may be involved in skeletal muscle regeneration. In this study, utilizing *Klf15* knockout (Klf15KO) mice, we analyzed the function of KLF15 in skeletal muscle regeneration. Klf15KO results in inefficient myoblast differentiation and delayed muscle regeneration in the cardiotoxin (CTX)-injured mice model (5, 15). Global profiling of the KLF15 regulated transcriptome revealed that the loss of KLF15 led to the downregulation of genes involved in cell differentiation and metabolic processes. Mechanistically, we found that FKBP5, a positive regulator of myoblast differentiation and a molecular link between stress and metabolic regulation (16, 17, 18), was directly recognized and induced by KLF15 during muscle regeneration. Finally, impaired muscle regeneration in Klf15KO mice was rescued from local delivery of FKBP5. Together, our data revealed a previously unrecognized effect of KLF15 on myoblast differentiation and muscle regeneration. Our results may inform novel treatment strategies for muscle disorders associated with injuries or diseases.

## Results

### Loss of KLF15 impairs skeletal muscle repair and regeneration upon injury

**Figure 1:**
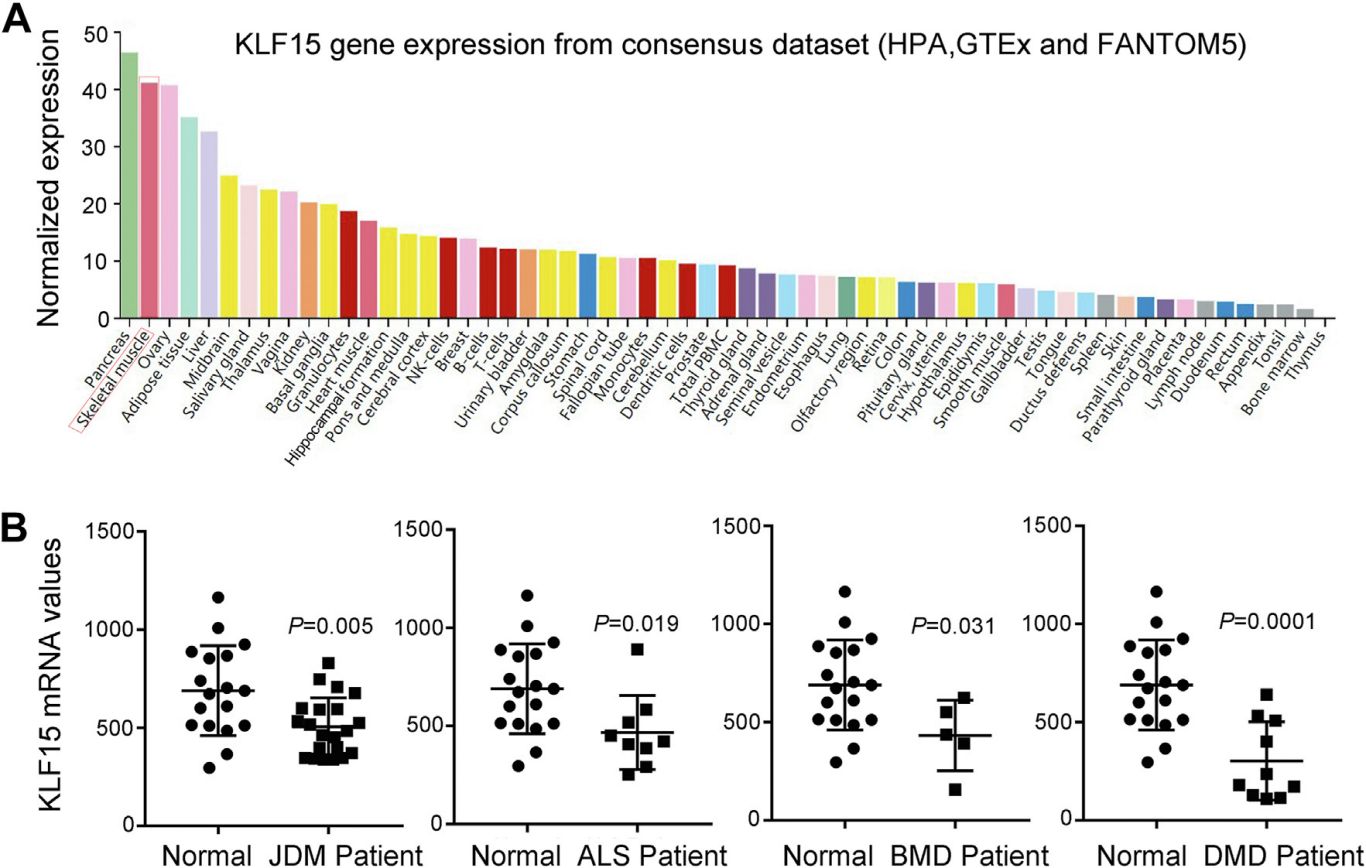
**Decreased levels of KLF15 in patients with muscle disease.***A*, KLF15 expression in human tissues using a public dataset (https://www.proteinatlas.org/ENSG00000163884-KLF15/tissue). *B*, expression of KLF15 in patients with skeletal muscle disease determined by querying the microarray datasets GSE3307. *p* value in two-tailed unpaired student *t* test are shown. ALS, amyotrophic lateral sclerosis; BMD, Becker muscular dystrophy; DMD, Duchenne muscular dystrophy; JDM, juvenile dermatomyositis; KLF15, Krüppel-like factor 15.

To identify the candidate genes potentially related to muscular diseases, the microarray dataset GSE3307 was downloaded from the Gene Expression Omnibus database. The downregulated molecule KLF15 was noticed because it was shown by the consensus dataset from HPA, GTEx, and FANTOM5 to be highly expressed in human skeletal muscle (Fig. 1*A*). However, KLF15 mRNA was significantly downregulated in patients with inflammatory degenerative muscle diseases, including JDM, ALS, BMD, and DMD (Fig. 1*B*). Because skeletal muscle possesses an extraordinary capacity for repair and regeneration to ensure healthy musculature, we hypothesized that KLF15 may play a role in skeletal muscle repair and regeneration after injury.

To test this hypothesis, we first generated Klf15KO mice by using the CRISPR/Cas9 system. Cas9 mRNA and two single guide RNAs targeting the sites near the translation start codon in exon 2 of *Klf15* were microinjected into mouse zygotes (Fig. S1*A*). Homozygous Klf15KO mice were born from heterozygous intercrosses. Successful elimination of Klf15 was confirmed by PCR genotyping (Fig. S1*B*), Sanger sequencing (Fig. S1*C*), and Western blot analysis of KLF15 protein expression in skeletal muscle tissue (Fig. S1*D*).

**Figure 2:**
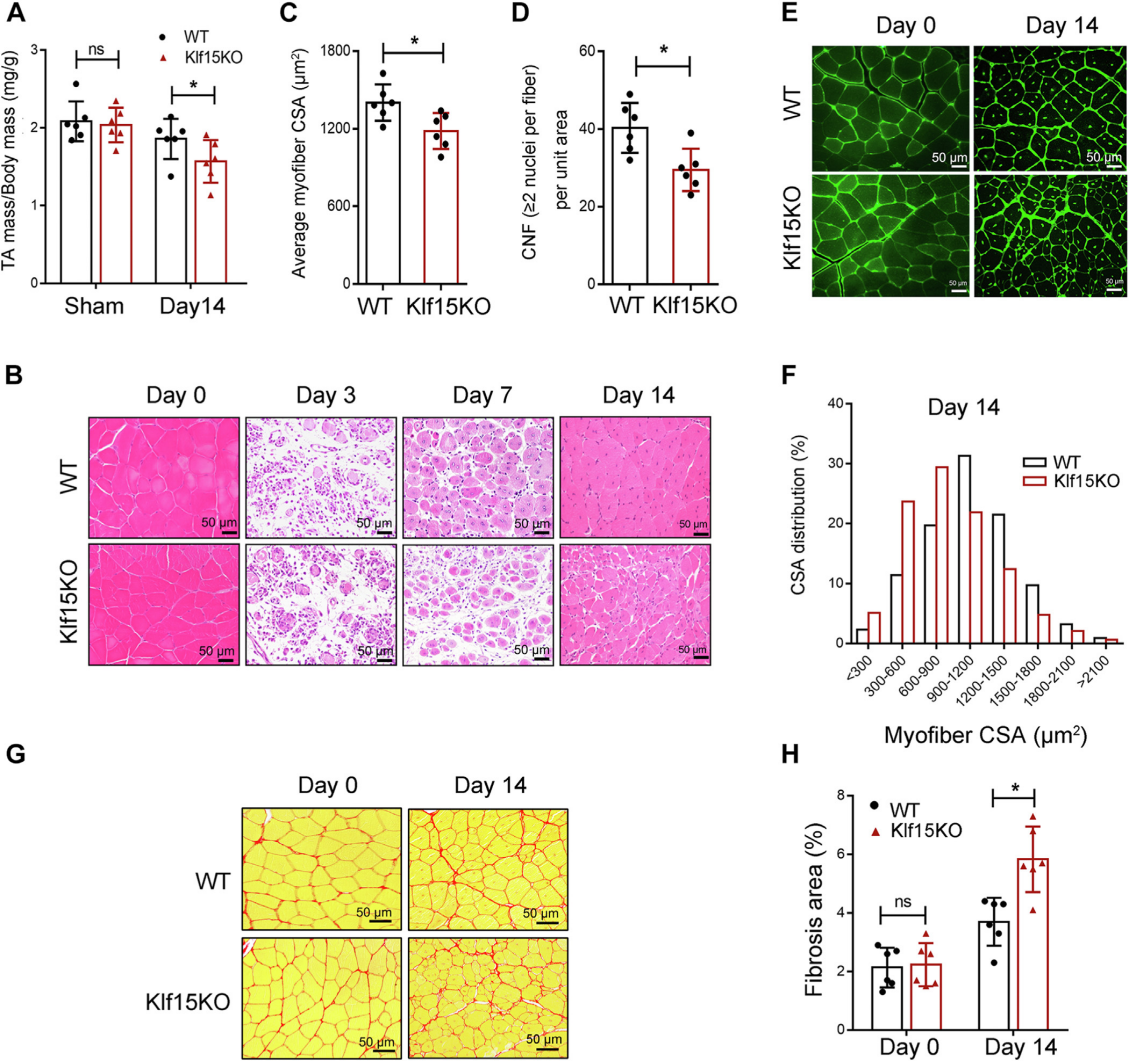
**Impaired muscle repair in Klf15KO mice upon injury.** CTX was injected into the TA muscles, and the mice were then sacrificed at different time points as indicated after injury. *A*, TA muscle weight normalized to body weight at day 14 after injury (n = 6, ∗*p* < 0.05, ns, no significant difference, unpaired student *t* test). *B*, representative H&E-stained TA muscles illustrating the course of regeneration in WT and Klf15KO mice (n = 4–6). *C*, quantification of average cross-sectional area (CSA) of regenerating myofibers at day 14 postinjury (n = 6, ∗*p* < 0.05, unpaired student *t* test). *D*, number of myofibers containing two or more centrally located nuclei per field at day 7 postinjury (n = 6, ∗*p* < 0.05, unpaired student *t* test). *E*, representative WGA-stained TA muscles from WT and Klf15KO mice illustrating the size of myofiber at day 14 post CTX injury (n = 6). *F*, the distribution of myofiber sizes in WT and Klf15KO mice was analyzed from the CSA of ∼250 myofibers of each sample. *G*, Picro-Sirius red–stained TA muscles showed the collagen deposition at days 0 and 14 after injury. *H*, quantitative analysis of the area of positive Picro-Sirius red staining in each group (n = 6, ∗*p* < 0.05, ns, no significant difference, unpaired student *t* test). CTX, cardiotoxin; KLF15, Krüppel-like factor 15.

Klf15KO mice were fertile and healthy and showed normal muscle development and histology under baseline physical conditions (Fig. S2). Although KLF15 deficiency alone does not cause any muscle pathology, we investigated whether KLF15 deficiency regulates the capacity for repair and regeneration in response to injury. To this end, we injected CTX into tibialis anterior (TA) muscles to induce injury, a well-established muscle regeneration model (5), and analyzed its capacity for recovery from injury. Fourteen days after injury, the decreased weight of TA muscle was observed in Klf15KO mice (Fig. 2*A*). Histologically, muscles in WT mice showed clear signs of injury, including inflammatory cell infiltration and degenerating fibers on day 3, and necrotic muscles were efficiently replaced by newly formed centrally nucleated fibers on day 7 postinjury. Fourteen days after injury, WT mice displayed uniformly sized, tightly packed muscle fibers (Fig. 2*B*). In contrast, TA muscles in Klf15KO mice showed poor signs of regeneration at days 7 and 14 following injury evident by the small myocytes (Fig. 2, *B*, *C*, and *E*), their fiber size distribution showed the leftward shift towards smaller fibers (Fig. 2*F*), the reduced number of newly formed fibers containing two or more centrally located nuclei (Fig. 2, *B* and *D*). Moreover, Picro-sirius red staining analysis of injured TA muscle on day 14 after injury revealed enhanced matrix accumulation (interstitial fibrosis) in Klf15KO mice (Fig. 2, *G* and *H*). The matrix accumulation observed in Klf15KO mice may be explained by the smaller newborn fibers. Collectively, we observed inefficient regeneration in Klf15KO skeletal muscle upon injury, suggesting an essential role for KLF15 in skeletal muscle repair under physiological stress.

### KLF15 is upregulated during myoblast differentiation

**Figure 3:**
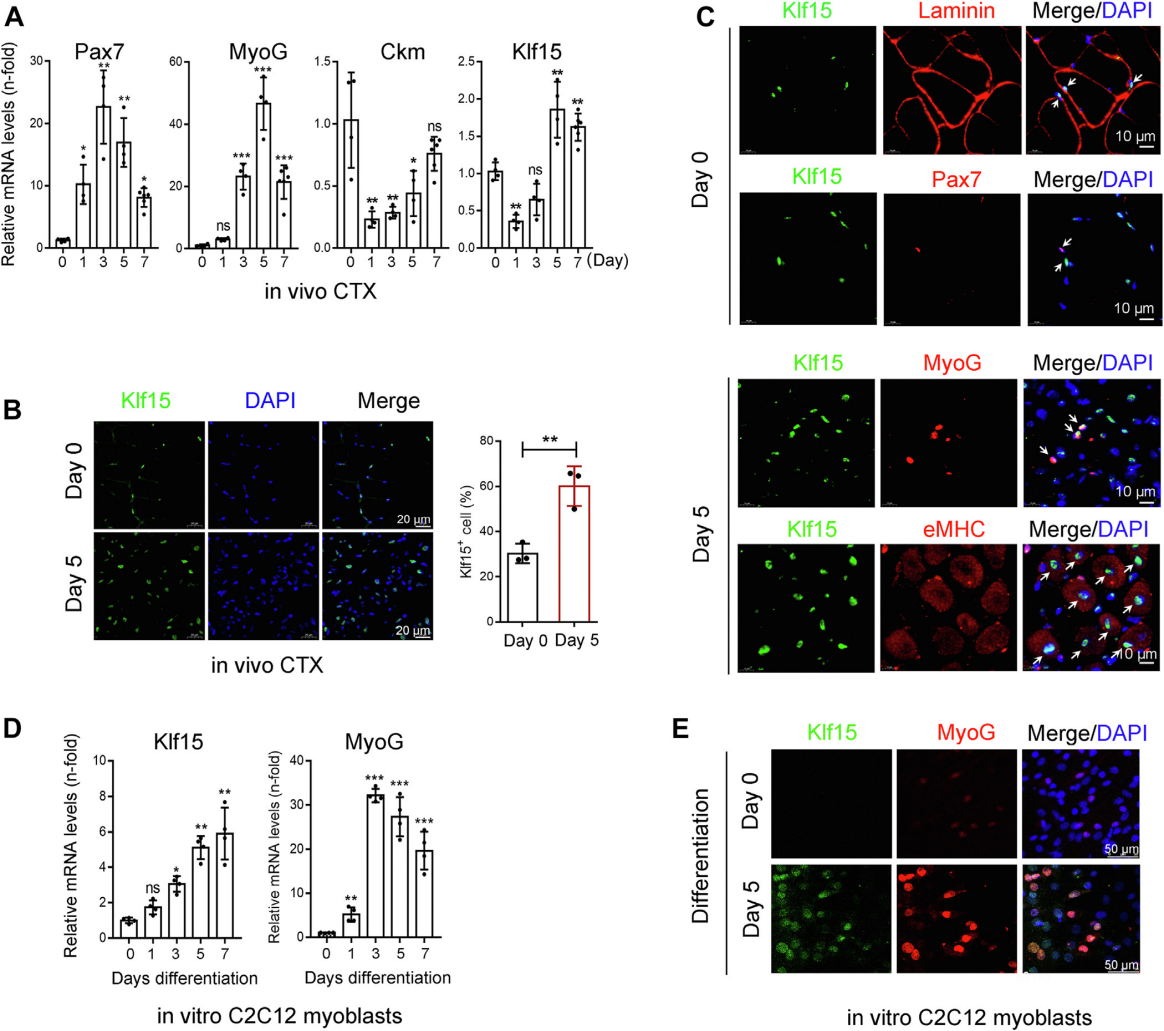
**KLF15 is upregulated during myoblast differentiation.***A*, relative mRNA levels of Pax7, MyoG, Ckm, and KLF15 in CTX-injured muscles at 0, 1, 3, 5, and 7 days postinjury were evaluated by qRT-PCR assays (n = 4–6, ∗*p* < 0.05, ∗∗*p* < 0.01, ∗∗∗*p* < 0.001, ns, no significant difference, one-way ANOVA). *B*, immunofluorescence for KLF15 (*green*) and percent of KLF15-positive cells in intact and CTX-injured TA muscles at day 5 (n = 3, ∗∗*p* < 0.01, unpaired student *t* test). *C*, co-immunofluorescence for KLF15 (*green*) with laminin (*red*) or Pax7 (*red*) in intact TA muscles and KLF15 (*green*) with MyoG (*red*) or eMHC (*red*) in CTX-injured TA muscles at day 5 (n = 3). *D*, C2C12 myoblasts were cultured in differentiation medium (2% horse serum), and the mRNA levels of KLF15 and MyoG were evaluated by qRT-PCR (n = 4, ∗*p* < 0.05, ∗∗*p* < 0.01, ∗∗∗*p* < 0.001, ns, no significant difference, one-way ANOVA). *E*, immunofluorescence staining for KLF15 (*green*) and MyoG (*red*) in undifferentiated and differentiated C2C12 cells (n = 4). CTX, cardiotoxin; eMHC, embryonic myosin heavy chain; KLF15, Krüppel-like factor 15; qRT-PCR, quantitative real-time PCR.

Muscle regeneration is a well-coordinated process that involves satellite cell activation and proliferation, myoblast differentiation, myofiber fusion, and growth (2, 19). To explore the mechanism of action of KLF15, we first examined its expression pattern of KLF15 during skeletal muscle regeneration. Upon CTX injury, muscle regeneration begins with the activation and proliferation of satellite cells (marked by the increased expression of Pax7^+^, at day 1 after injury), followed by myoblast differentiation (early stage, marked by the induction of MyoG, at day 3–5 post injury) and ultimately, myofiber fusion and growth (late stage, marked by the expression of Ckm, on day 7–14) as shown here and other studies (Fig. 3*A*). We found that KLF15 mRNA was downregulated on day 1 after injury and then upregulated between days 5 and 7 (Fig. 3*A*). The increased expression of KLF15 was confirmed by immunofluorescence staining of TA muscle on day 5 after CTX injection (Fig. 3*B*). To clarify the expression of KLF15 during myogenesis, we performed double immunofluorescence on serial sections of TA muscles where KLF15 was costained with laminin or Pax7 at day 0 and costained with MyoG or embryonic myosin heavy chain (eMHC) at day 5 after CTX treatment. KLF15 protein was detected in mature myofiber and was undetectable in Pax7^+^ quiescent satellite cells. However, KLF15 protein was highly expressed in MyoG^+^ muscle progenitors and in eMHC^+^-differentiated myogenic cells (Fig. 3*C*). We also confirmed the upregulation of KLF15 during myoblast differentiation *in vitro* using C2C12 myoblast, a well-established model for studying myoblast differentiation (20, 21). Indeed, KLF15 expression was significantly upregulated in C2C12 cells undergoing myogenic differentiation, but barely detectable in proliferating C2C12 cells (Differentiation Day 0), as indicated by both quantitative real-time PCR (qRT-PCR) and immunofluorescent staining assays (Fig. 3, *D* and *E*). These data indicated increased KLF15 expression during myogenic differentiation.

### KLF15 deficiency impairs myoblast differentiation and myotube maturation

**Figure 4:**
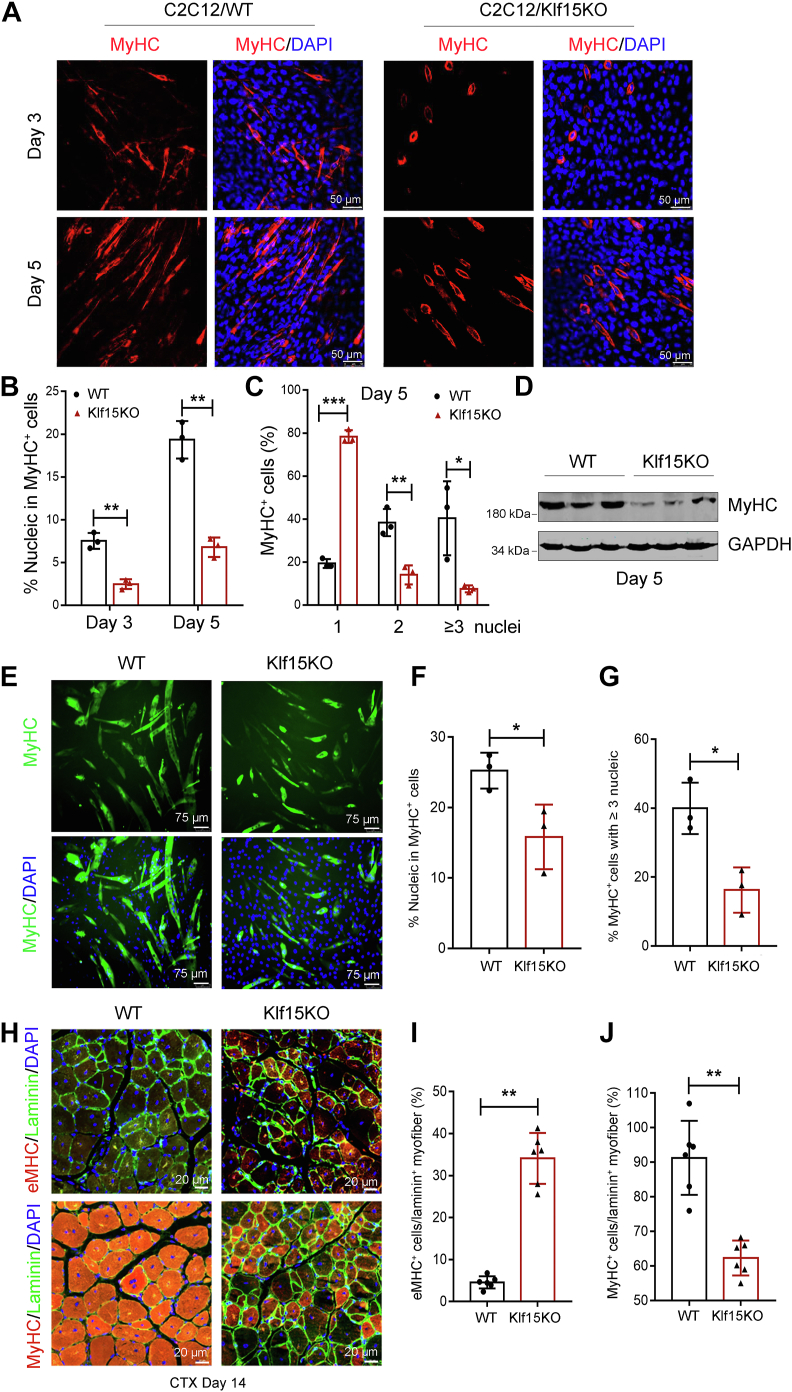
**KLF15 deficiency results in inefficient myoblast fusion and myotube maturation *in vitro* and *in vivo*.***A*–*D*, WT or Klf15-deficient C2C12 myoblasts (constructed using a CRISPR-Cas9 system) were incubated with differentiation medium for 3 or 5 days. Representative images of MyHC immunofluorescence (*red*) (*A*), quantification of MyHC^+^ cells per field (*B*), the frequency of MyHC^+^ cells containing the indicated number of nuclei (*blue*) (*C*), and MyHC protein levels (*D*). Data shown are representative of three independent experiments (n = 3, ∗*p* < 0.05, ∗∗*p* < 0.01, ∗∗∗*p* < 0.001, unpaired student *t* test). *E*–*G*, primary myoblasts derived from the muscles of WT and Klf15KO mice were incubated in differentiation medium for 5 days followed by staining for MyHC (*green*) and DAPI (*blue*). Representative images of MyHC immunofluorescence (*E*), quantification of MyHC^+^ cells per field (*F*), the frequency of MyHC^+^ cells containing ≥ 3 nucleic per field (*G*) (n = 3, ∗*p* < 0.05, unpaired student *t* test). *H*, immunostaining for eMHC (*red*) or MyHC (*red*) and laminin (*green*) in WT and Klf15KO TA muscles at day 14 after CTX injection. Percentage of eMHC^+^ fibers per laminin^+^ myofiber (*I*) or MyHC^+^ fibers per laminin^+^ myofiber (*J*) were quantified in WT and Klf15KO muscles (n = 6, ∗∗*p* < 0.01, unpaired student *t* test). eMHC, embryonic myosin heavy chain; KLF15, Krüppel-like factor 15.

Consistent with the high expression of KLF15 in differentiated but not proliferated myogenic cells, KLF15 had no effect on the activation and proliferation of satellite cells, as evidenced by the similar numbers of PAX7^+^ cells in WT and Klf15KO mice on day 5 after injury (Fig. S3). The robust expression of KLF15 during myoblast differentiation (Fig. 3, *C*–*E*) prompted us to investigate whether KLF15 affects myoblast fusion and myotube maturation. To this end, KLF15-null C2C12 cells were generated using the CRISPR-Cas9 system (22). Deletion of KLF15 was confirmed by DNA sequencing and Western blot analysis (Fig. S4). We then analyzed the role of KLF15 in myoblast fusion by myosin staining of C2C12 cells on days 3 and 5 after differentiation. As shown in Figure 4*A*, myoblasts deficient in KLF15 displayed a marked reduction in their fusion capacity. In particular, the number of nuclei within the mature myosin heavy chain positive (MyHC^+^) myotubes was significantly reduced in Klf15-null cells (Fig. 4*B*). Moreover, 79% of the MyHC^+^ cells in the control group fused to myotubes (one MyHC^+^ cell with at least two nuclei) but only 22% of the MyHC^+^ cells in the Klf15-null group fused to myotubes on day 5 after differentiation (Fig. 4*C*), suggesting that KLF15 deficiency impairs myoblast fusion *in vitro*. Consistently, loss of KLF15 significantly reduced the levels of MyHC protein (Fig. 4*D*). Moreover, we found that the ability of primary myoblasts derived from the muscles of Klf15KO mice to fuse to myotubes was decreased significantly (Fig. 4, *E*–*G*), which was consistent with the results of KLF15-null C2C12 cells. Finally, we evaluated myofiber maturation in Klf15KO muscles *in vivo*. Immunofluorescence staining was performed to detect the immature eMHC-positive (eMHC^+^) fibers and mature MyHC^+^ fibers in the 14-days–injured TA muscle sections (6). Fibers in Klf15KO mice maintained strong eMHC signals, and the number of mature MyHC^+^ muscle fibers was reduced compared to WT mice (Fig. 4, *H*–*J*), providing evidence that Klf15KO impedes muscle maturation following injury. Thus, our data suggested that KLF15 plays an essential role in the late stages of skeletal muscle regeneration.

### Klf15KO muscles decrease regeneration and metabolic processes and activate extracellular matrix remodeling processes

**Figure 5:**
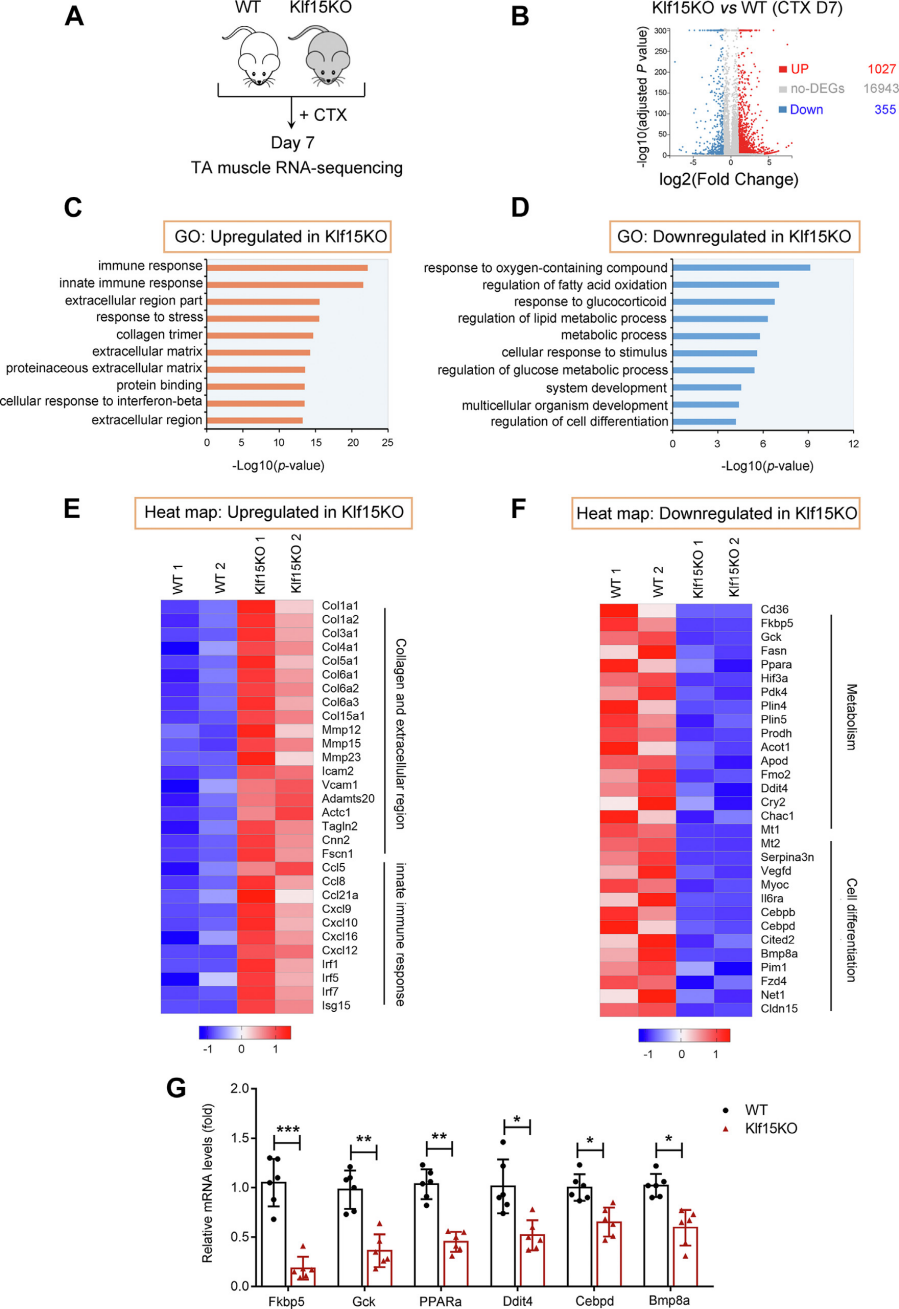
**Klf15KO muscles decrease cell differentiation and metabolic processes.***A*, the transcription expression profiling of TA muscle from WT and Klf15KO mice at day 7 after CTX injection were accessed by RNA-seq (n = 3). *B*, the scatter plot showing differentially expressed genes (DEGs). *C* and *D*, functional annotations of upregulated and downregulated genes in Klf15KO mice, respectively. *E*, heatmap of genes upregulated in Klf15KO muscle related to collagen, extracellular region, and innate immune response. *F*, heatmap of genes downregulated in Klf15KO muscle related to metabolism and cell differentiation. *G*, qRT-PCR verified the mRNA levels of the candidate genes categorized into metabolism and cell differentiation in WT and Klf15KO muscles at day 7 postinjury (n = 6, ∗*p* < 0.05, ∗∗*p* < 0.01, ∗∗∗*p*< 0.001, unpaired student *t* test). CTX, cardiotoxin; KLF15, Krüppel-like factor 15; qRT-PCR, quantitative real-time PCR.

To understand why KLF15 deficiency inhibits muscle regeneration, transcriptome analysis was performed on TA muscles isolated from WT and Klf15KO mice on day 7 after CTX injection (Fig. 5*A*). Compared to the control, 1382 DEG, including 1027 upregulated genes and 355 downregulated genes, were identified in Klf15KO muscle after CTX injection (Fig. 5*B*, Tables S1 and S2). Gene Ontology enrichment analysis showed that KLF15 deficiency–induced upregulated genes were mainly involved in the innate immune response, collagen trimer, and extracellular matrix (ECM) (Fig. 5, *C* and *E*), supporting an increased ECM remodeling process. Downregulated genes were dominated by metabolic processes, system development, and cell differentiation (Fig. 5, *D* and *F*). Finally, using qRT-PCR, we confirmed six candidate genes from the downregulated list with significantly decreased expression in Klf15KO muscles (Fig. 5*G*). Together, the loss of KLF15 leads to decreased differential processes and increased ECM remodeling.

### KLF15 transactivates FKBP5

**Figure 6:**
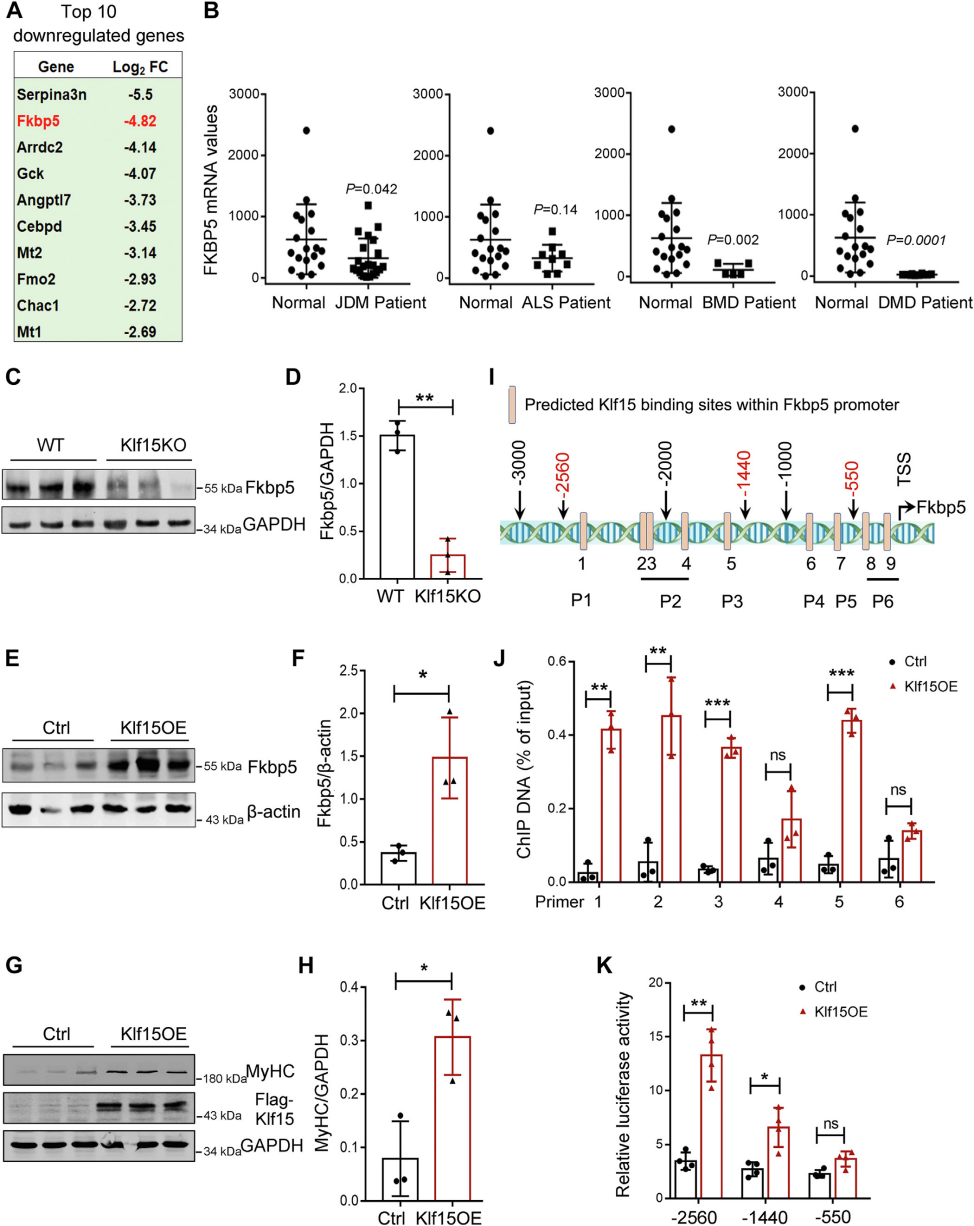
**Transcriptional regulation of FKBP5 by KLF15.***A*, top ten downregulated genes in Klf15KO muscles at day 7 after CTX injection. Average FPKM in WT muscle >10; log2(Klf15KO/WT) ≤−1; adjusted *p* value < 0.001. *B*, expression of FKBP5 in patients with skeletal muscle disease determined by querying the GSE3307 datasets. *p* value in two-tailed unpaired student *t* test are shown. *C* and *D*, protein levels of FKBP5 in WT and Klf15KO muscles at day 7 postinjury were quantified using Western blot and normalized to GAPDH (n = 3, ∗∗*p* < 0.01, unpaired student *t* test). *E* and *F*, protein levels of FKBP5 in control and Klf15-overexpressed (Klf15OE) C2C12 cells were quantified using Western blot and normalized to β-actin (n = 3, ∗*p* < 0.05, unpaired student *t* test). *G* and *H*, protein levels of MyHC in control and Klf15-overexpressed (Klf15OE) C2C12 cells under differentiation for 5 days were quantified using Western blot and normalized to GAPDH (n = 3, ∗*p* < 0.05, unpaired student *t* test). Overexpression of KLF15 was confirmed by an anti-Flag antibody. *I*, mapping of the previously reported KLF15-binding sites (CACCC, GGGGCG, and GGGGNGGNG) in mouse FKBP5 promoter. P1-P6, Primer 1 to 6 used in (*J*). *Red* numbers indicate the sequences used in the reporter constructs in (*K*). *J*, recruitment of KLF15 onto mouse FKBP5 promoter. C2C12 cells were transfected with Flag-KLF15 or pcDNA control plasmid and subjected to ChIP by using an anti-Flag antibody. ChIP DNA was shown as percentage of input DNA (n = 3, ∗∗*p* < 0.01, ∗∗∗*p* < 0.001, ns, no significant difference, unpaired student *t* test). *K* relative luciferase activity of C2C12 myoblasts transfected with the reporter constructs with or without KLF15 overexpression (n = 4, ∗*p* < 0.05, ∗∗*p* < 0.01, ns, no significant difference, unpaired student *t* test). ALS, amyotrophic lateral sclerosis; BMD, Becker muscular dystrophy; ChIP, chromatin immunoprecipitation; CTX, cardiotoxin; DMD, Duchenne muscular dystrophy; FKBP5, FK506-binding protein 51; JDM, juvenile dermatomyositis; KLF15, Krüppel-like factor 15.

To define the molecular targets mediating Klf15 action, we screened genes in the list of DEGs critical for muscle repair and regeneration and found that FKBP5 was ranked as one of the most strongly downregulated genes in Klf15KO muscle on day 7 after injury (Fig. 6*A*). Importantly, by querying the GSE3307 database, we found that skeletal muscle FKBP5 mRNA was downregulated in patients with JDM, BMD, and DMD (Fig. 6*B*), which also showed downregulation of KLF15 compared with healthy controls (Fig. 1*B*). FKBP5 is a peptidyl-prolyl isomerase that has recently been identified as a critical regulator of myoblast differentiation and myotube formation (16, 17). Therefore, FKBP5 was selected for further study. The significant downregulation of FKBP5 in injured Klf15KO muscle was further confirmed by qRT-PCR and Western blot analysis (Figs. 5*G* and 6, *C* and *D*). Conversely, KLF15 overexpression induced high levels of FKBP5 expression in C2C12 cells *in vitro* (Fig. 6, *E* and *F*). A reciprocated result of overexpression of Klf15 on differentiation of myoblasts was evidenced by the increased protein levels of MyHC at day 5 after differentiation (Fig. 6, *G* and *H*). We then investigated whether FKBP5 is a direct target of KLF15. To address the role of KLF15 in the transcriptional regulation of FKBP5, we searched for the previously reported KLF15 consensus binding sequence CACCC and GC-rich motif (GGGGCG and GGGGNGGNG) (23) in the promoter region of FKBP5 (−3000 to +1). We identified that the FKBP5 promoter was highly enriched in the KLF15 consensus-binding motif (Fig. 6*I*). We then performed a chromatin immunoprecipitation (ChIP) assay to confirm the binding of KLF15 to the promoter region of FKBP5. KLF15 binds to multiple sites in the FKBP5 promoter region (Fig. 6*J*). To further confirm the transcriptional activation of FKBP5 by KLF15, we performed a reporter assay where the constructs carrying the regulatory promoter regions of Fkbp5 were cotransfected into C2C12 cells with KLF15 plasmid. Overexpression of KLF15 increased the Fkbp5 promoter-luciferase reporter. The deletion of Fkbp5 promoter indicated that the region from −2560 to −550 in Fkbp5 promoter was functional (Fig. 6*K*). Thus, our data indicated that KLF15 transactivates FKBP5.

### Impaired muscle regeneration of KLF15-deficient mice is rescued by FKBP5

**Figure 7:**
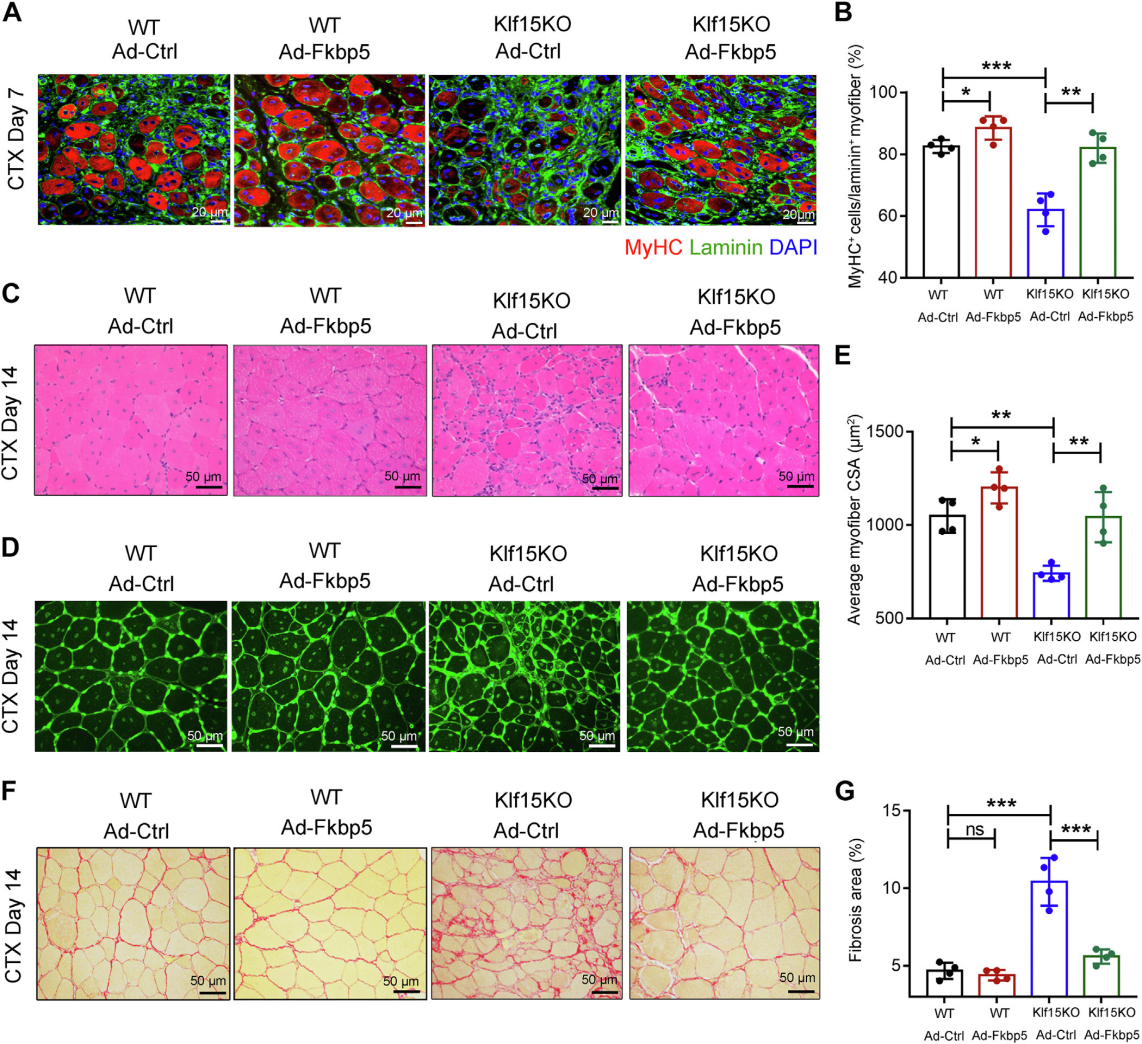
**FKBP5 rescues Klf15KO mice from impaired muscle regeneration.** TA muscles of WT and Klf15KO mice were injected with adenovirus expressing FKBP5 (Ad-FKBP5) or control adenovirus (Ad-Ctrl) at day 1 post-CTX injury and harvested at day 7 or day 14 after injury. *A*, immunostaining for MyHC (*red*) and laminin (*green*) in TA muscles at day 7. *B*, percentage of MyHC^+^ fibers per laminin^+^ myofiber were quantified (n = 6, ∗*p* < 0.05, ∗∗*p* < 0.01, ∗∗∗*p* < 0.001, one-way ANOVA). *C*, representative H&E-stained TA muscles at day 14 (n = 6). *D*, representative WGA-stained TA muscles illustrating the size of myofiber at day 14. *E*, quantification of average cross-sectional area (CSA) of regenerating myofibers at day 14 (n = 6, ∗*p* < 0.05, ∗∗*p* < 0.01, one-way ANOVA). *F*, Picro-Sirius red–stained TA muscles showed the collagen deposition at day 14. *G*, quantitative analysis of the area of positive Picro-Sirius red staining (n = 6, ∗∗∗*p* < 0.001, ns, no significant difference, one-way ANOVA). CTX, cardiotoxin; FKBP5, FK506-binding protein 51; KLF15, Krüppel-like factor 15.

We then attempted to rescue the delayed muscle repair and regeneration in Klf15KO muscles by local delivery of FKBP5. We injected adenovirus carrying FKBP5 (Ad-FKBP5) or control adenovirus (Ad-Ctrl) into the TA muscles of WT and Klf15KO mice on day 1 after CTX injection and harvested TA muscles to examine regeneration on days 7 and 14 after CTX injury. Enhanced expression of FKBP5 after viral infection was confirmed by Western blot and immunohistochemical staining (Fig. S5). We observed enhanced muscle regeneration in the Ad-FKBP5–treated WT and Klf15KO mice, as evidenced by an increased number of newly formed MyHC^+^ myofibers on day 7 (Fig. 7, *A* and *B*), an increased size of myocytes, a reduction in necrotic fibers, and a reduction in fibrosis area on day 14 (Fig. 7, *C*–*G*). Thus, our data suggest that the local delivery of FKBP5 promotes muscle repair and rescues Klf15-deficient mice from impaired muscle regeneration.

## Discussion

The downregulation of KLF15 in patients with various muscle diseases, including JDM, ALS, BMD, and DMD, prompted us to investigate whether KLF15 is involved in muscle repair and regeneration, a unique adaptation of the muscle in response to injury or disease. Using the Klf15KO mouse model, we observed that KLF15 deficiency delayed the process of regeneration in CTX-induced muscle injury, suggesting the essential functions of KLF15 in regulating skeletal muscle regeneration. KLF15 is a metabolic transcription factor that regulates glucose, amino acid, and lipid utilization in muscle. KLF15-dependent metabolic gene program increases endurance exercise capacity in mice and attenuates features of disease severity in the mdx mouse model of DMD (24, 25). In this study, we investigated the role of KLF15 in muscle injury to promote muscle regeneration.

The differentiation of satellite cells into multinucleated myofibers is governed by myogenic regulatory factors (MRFs) and various intracellular signaling molecules (1). KLF15-deficient mice develop normal skeletal muscles, and loss of KLF15 has no effect on satellite cell activation upon injury, suggesting that KLF15 is dispensable for skeletal muscle development and does not directly regulate MRFs. Consistently, increased KLF15 expression was observed after induction with Pax7 and MyoD. Although KLF15 does not directly target MRFs, when introduced into an acute injury model, this mouse strain showed inefficient myoblast differentiation, suggesting that KLF15 may target intracellular signaling molecules involved in myoblast differentiation and myofiber formation. Unbiased transcriptomic profiling confirmed the dominant downregulation of genes involved in cell differentiation. ChIP assay identified FKBP5 as a KLF15 target. Finally, impaired muscle regeneration in Klf15KO mice was rescued by local delivery of FKBP5.

FKBP5 is a widely expressed peptidyl-prolyl isomerase that serves as a molecular chaperone of the glucocorticoid receptor complex and functions as a molecular link between stress and metabolic regulation (18, 26). FKBP5 promotes myogenic differentiation *via* two different mechanisms. One mechanism is cell cycle regulation, FKBP5 sequesters cyclin-dependent kinase 4 (Cdk4) in the Hsp90 complex and inhibits Cdk4 phosphorylation, thus facilitating the proliferating myoblasts to exit from the cell cycle and fuse to form myofibers (16). The high activity of Cdk4 in myotonic dystrophy cells during all stages of differentiation leads to impaired cell cycle withdrawal and muscle differentiation (27). The other mechanism is metabolic reprogramming. It is clear that the energetic demands during the regeneration process (activation, proliferation, differentiation, and self-renewal of satellite cells) are likely to be very different. Two major metabolic pathways provide energy for cells: oxidative phosphorylation (OXPHOS) and glycolysis. OXPHOS is the most efficient producer of ATP during aerobic respiration, generating an average of 36 ATP molecules per glucose. In contrast, glycolysis is relatively inefficient for ATP production, but it can generate ATP rapidly in response to acute changes in energy demand and provide glycolytic intermediates that are critical for the synthesis of macromolecules, such as amino acids, lipids, and nucleic acids, which are important for the activation and replication of satellite cells. Thus, early activation of satellite cells may rely more on glycolysis than OXPHOS for energy production and then shift from glycolysis to OXPHOS during late differentiation and fusion (28, 29, 30, 31). Decreased FKBP5 expression has been shown to impair mitochondrial OXPHOS and inhibit myogenic differentiation (17). Collectively, the functions of FKBP5 on both cell cycle exit and OXPHOS indicate a positive regulation in the late differentiation and fusion, but not in the early activation and proliferation of satellite cells. Consistently, KLF15 deficiency impairs myoblast differentiation and myofiber maturation but has no effect on the activation and proliferation of satellite cells.

It is well known that glucocorticoid (GC) steroids are widely used to treat many chronic diseases, including muscle disease (32, 33). The functions of GC steroids to mediate ergogenic muscle performance effects are shown to rely on KLF15 (24). Interestingly, the most downregulated genes in Klf15KO muscle after CTX injection, including Serpina3n and metallothionein 2 (Fig. 6*A*), are also known direct targets of glucocorticoid receptors (34, 35). Serpina3n has a protective role towards skeletal muscle in both mdx models and acute muscle injury by its antiprotease activity towards extracellular proteases to reduce destructive protease activity and maintain the regenerative capacity of skeletal muscle (36). Therefore, the cooperative regulation of these protective genes by the two transcription factors KLF15 and glucocorticoid receptor may confirm the therapeutic role of the GC–KLF15 axis in skeletal muscles.

Although KLF15 deficiency is harmful to skeletal muscle, leading to delayed muscle regeneration upon injury as shown here or exaggerated muscle damage in mdx mice as suggested by other group, several lines of evidence suggest that aberrant KLF15 expression on myofibers and myocytes may also contribute to the regulation of muscle atrophy–related genes Atrogin1 and Murf1 and dystrophy pathology (23, 37). Thus, it would be interesting to clarify in future studies how KLF15 balances differentiation and muscle dysfunction in different pathological microenvironments. Our findings extend the role of KLF15 in muscle injury to promote muscle regeneration and highlight the therapeutic potential of targeting KLF15 in muscle disorders.

## Experimental procedures

### Animals

We used 8 to 12-week-old male C57BL/6J mice. They had age-matched littermate controls and were bred and housed in pathogen-free conditions at the animal facilities of Beijing Anzhen Hospital affiliated with the Capital Medical University (Beijing). Animal experimentation was approved by the Animal Subjects Committee of Capital Medical University.

Klf15KO mice were generated using the CRISPR/Cas9 system. Briefly, Cas9 mRNA and two guide RNAs targeting sites upstream and downstream of exon 2 of *Klf15* were microinjected into the fertilized embryos of C57BL/6J mice. Klf15KO founders were identified using PCR and Sanger sequencing. Homozygous Klf15KO mice were born from a heterozygous intercross and used for phenotypic analysis. The guide RNAs sequences are listed in Table S3.

### Skeletal muscle injury

A CTX injury–induced muscle regeneration model was used in this study and was established as previously described (15, 38). Briefly, anesthetized mice were injected with 30 μl of 10 μM CTX (Sigma) into the TA muscle. Regenerating muscles were isolated 1, 3, 5, 7, and 14 days after CTX injection and prepared for histological analysis.

### Tissue preparation, histology, and immunostaining

The TA muscles were collected, fixed, and processed in paraffin or frozen sections. For paraffin sectioning, TA muscles were fixed with 4% paraformaldehyde at 25 °C for 6 h and processed for routine paraffin histology. For frozen sections, TA muscles were mounted in optimum cutting temperature compound, immediately frozen in cooled isopentane chilled with liquid nitrogen, and stored at −80 °C. Tissues were cut into 7-μm-thick cross-sections for further studies. H&E staining, Picro-sirius red staining, and FITC-conjugated wheat germ agglutinin staining were carried out using standard procedures, as reported previously (15). Images were captured using an Eclipse 90i digital microscopy system (Nikon) and analyzed using the NIS-Elements Br 3.0.

For immunofluorescence, frozen sections of the muscle were fixed with 4% paraformaldehyde, permeabilized with 0.3% Triton X-100, and blocked with 10% goat serum. After washing with PBS, the slides were incubated with the primary antibody at 4 °C for 16 to 18 h, followed by incubation with the secondary antibody donkey anti-mouse IgG (Fluor Alexa 488, Thermo Fisher Scientific) for 1 h, and mounted with DAPI mounting medium (Zhongshan Golden Bridge Biotechnology). Images were captured using an Olympus fluorescence microscope. The primary antibodies used for immunostaining are listed in Table S4.

### Primary myoblasts isolation and C2C12 myoblasts culture, differentiation, and immunostaining

Primary myoblasts were isolated and cultured with a modified protocol (39). Briefly, the hind limb muscles of 3-week-old WT and Klf15KO mice were digested by collagenase/dispase/CaCl_2_ solution at 37 °C for about 30 min until the mixture is a fine slurry. The slurry was then sequentially passed through a 70-μm and then 30-μm cell strainer (BD Falcon). The filtrate was centrifuged and resuspended in F-10–based primary myoblast growth medium and plated in a collagen-coated culture dish. The medium was changed every 2 days. The cells were removed from the dish using PBS with no trypsin or EDTA and were preplated on a collagen-coated dish for 15 min to leave fibroblasts. When the fibroblasts are no visible in the culture, the medium was changed to F-10/Dulbecco’s modified Eagle’s medium (DMEM)-based primary myoblast growth medium. C2C12 myoblasts were purchased from the cell bank of the National Experimental Cell Resource Sharing Platform and maintained in growth medium (DMEM supplemented with 10% fetal bovine serum and 1% penicillin-streptomycin). To induce primary or C2C12 myoblasts differentiation into myotubes, the culture medium was replaced with differentiation medium (DMEM with 2% horse serum and 1% penicillin-streptomycin) when the cells were approximately 80% confluent. The cells were supplemented with fresh differentiation medium every 24 h for 5 to 7 days.

To generate Klf15KO C2C12 cells, px459 plasmids carrying the CRISPR-Cas9 expression cassette and guide RNA targeting exon 2 of *Klf15* were transfected into C2C12 cells using the P5 Primary Cell 4D-Nucleofector X Kit (LONZA) following the manufacturer’s instructions. The transfected cells were selected using puromycin, and the deletion of KLF15 was confirmed using Sanger sequencing.

For immunostaining of myotubes, C2C12 myoblasts or primary myoblasts were grown on culture slides (BD Biosciences) in a differentiation medium for 5 days. The cells were then fixed with 4% paraformaldehyde for 10 min, permeabilized with 0.3% Triton X-100 for 5 min, and blocked with 10% goat serum for 30 min. The cells were incubated with an anti-MyHC antibody (1:50; clone MF20; DSHB) at 4 °C for 16 to 18 h. After washing with PBS, the culture slides were incubated with the secondary antibody donkey anti-mouse IgG (Fluor Alexa 594, Thermo Fisher Scientific) for 1 h at 25 °C and mounted with DAPI mounting medium (Zhongshan Golden Bridge Biotechnology). Cells were observed under an Olympus fluorescence microscope.

### RNA-seq and data analysis

WT and Klf15KO mice were injected with CTX into TA muscles for 7 days. TA muscles were collected for RNA extraction using TRIzol reagent (Invitrogen). The quality of the total RNA was evaluated using a NanoDrop spectrophotometer (Thermo Fisher Scientific). Library construction and RNA-seq were performed using the BGISEQ-500 RNA-Seq platform (Beijing Genomic Institution; www.genomics.org. cn, BGI). DEGs were defined using the bioinformatics service of BGI according to the combination of the absolute value of log_2_-fold change >1 and adjusted *p*-value (FDR) ≤0.001 (40).

### Quantitative real-time PCR

Total RNA was isolated from TA muscles or C2C12 myoblasts using TRIzol reagent (Thermo Fisher Scientific). Total RNA concentration was determined using Qubit 4 (Thermo Fisher Scientific). Purified RNA (2 μg) was subjected to reverse transcription using Moloney murine leukemia virus reverse transcriptase (Promega). Expression of the gene of interest was determined using SYBR Green-based qRT-PCR (Takara), performed on a BIORAD iCycler iQ5 (Bio-Rad). Primer sequences used for qRT-PCR are listed in Table S3. Gene expression levels were calculated from threshold cycle (Ct) values using the 2^−△△Ct^ method, with GAPDH as an internal control.

### Western blot analysis

Muscle proteins were extracted using the Tissue Protein Extraction Reagent with the addition of a protease inhibitor cocktail (Thermo Fisher Scientific). After incubation on ice for 30 min, samples were centrifuged at 12,000 rpm for 15 min at 4 °C. The supernatants were collected and concentrated using a BCA protein assay kit (Thermo Fisher Scientific), following the manufacturer’s instructions. Approximately, 50 μg of protein was loaded and separated using SDS-PAGE. Proteins were transferred onto polyvinylidene difluoride membranes. After blocking with 5% bovine serum albumin, the membrane was incubated with the primary antibody for 16 to 18 h at 4 °C, followed by incubation with infrared dye 800CW-conjugated secondary antibodies (Li-COR Biosciences) for 2 h at 25 °C. Bands were detected using an Odyssey infrared imaging system (LI-COR Biosciences). The primary antibodies used for Western blot analysis are listed in Table S4.

### In silico promoter analysis

The genomic sequence of mouse FKBP5 (ID:14229) was downloaded from GenBank and analyzed using SnapGene software (https://www.snapgene.com/). Previously identified KLF15-binding sequences, CACCC, and GC-rich motifs (GGGGCG and GGGGNGGNG) (23) were searched in the promoters of FKBP5 (−3000 to +250) to identify putative KLF15-binding sites.

### ChIP assay

ChIP assays were performed in differentiated C2C12 cells overexpressing Flag-KLF15 using the SimpleChIP Enzymatic Chromatin IP Kit (Agarose beads, #9002, Cell Signaling Technology), following the manufacturer’s instructions. Briefly, approximately 8 × 10^6^ cells were fixed in formaldehyde. Chromatin was fragmented using micrococcal nuclease digestion and subjected to immunoprecipitation using an anti-Flag antibody, with histone H3 antibody as the positive control and mouse IgG as the negative control. After chromatin immunoprecipitation, the bound fraction and input DNA were subjected to qRT-PCR. Primers used are listed in Table S3.

### Luciferase reporter assay

C2C12 cells were cotransfected with pcDNA or pcDNA-Flag-KLF15 expression plasmid, a luciferase reporter carrying the different promoter regions of Fkbp5 (−2560 to +1; −1440 to +1; −550 to +1) and the internal control pRL-TK. At 36 h post-transfection, luciferase activity was measured using the dual-luciferase reporter assay system (Promega) according to the manufacturer’s recommendations.

### FKBP5 gene transfer

To test the effect of FKBP5 overexpression in the rescue of muscle regeneration in Klf15KO mice, 30 μl (4 × 10^9^ PFU) of adenovirus expressing FKBP5 (Ad-FKBP5) or Ad-GFP was injected into the TA muscle at day 1 after CTX injury. The TA muscle was collected and analyzed on days 7 and 14 after the CTX injection.

### Statistical analysis

Data are shown as mean ± SD of at least three replicates. Statistical significance was calculated using an unpaired student *t* test for comparisons between two groups or one-way ANOVA followed by Tukey’s test for post hoc comparison to assume differences among more than two groups. *p* < 0.05 is considered statistically significant.

## Data availability

All data generated during this study are included in this published article and its supporting information files.

## Supporting information

This article contains supporting information.

## Conflict of interest

The authors declare that they have no conflicts of interest with the contents of this article.
