# Supplementary material for: The transcriptional regulator KLF15 is necessary for myoblast differentiation and muscle regeneration by activating FKBP5
Source: J Biol Chem. 2023 Sep 4;299(10):105226. doi: 10.1016/j.jbc.2023.105226 (PMC10622842; doi:10.1016/j.jbc.2023.105226)
Supplement: Supporting Tables S1–S4 [file mmc1.pdf]

Table S1 Upregulated genes in Klf15KO muscles at Day 7 after CTX injection

Gene	WT CTX D7 Average FPKM	Klf15KO CTX D7 Average FPKM	log2 (Klf15KO/WTD7)	Qvalue (WT-vs-Klf15KO)	Pvalue (WT-vs-Klf15KO)
Gm9840	0	5.9	8.12	4.86E-30	1.66E-30
Gm21541	0	2.5	7.69	1.31E-23	5.45E-24
Ccl21c	0	2.5	7.69	1.31E-23	5.45E-24
Hbb-b1	0.2	42.6	7.53	1.02E-266	4.55E-268
Rps27rt	0	5.1	7.4	5.15E-20	2.40E-20
Hist1h2ao	0	1.6	6.37	4.15E-11	2.95E-11
Hbb-b2	0.1	4.1	6.24	3.83E-29	1.34E-29
Prnd	0.2	11.5	5.69	0	0
Bglap2	0	2.3	5.6	6.80E-13	4.37E-13
Cenpm	0.1	1.3	4.93	1.68E-15	9.56E-16
Gm3646	0.1	1.9	4.91	2.14E-08	1.81E-08
Apln	0.1	2.4	4.81	3.40E-84	4.64E-85
Mxd3	0.1	1.4	4.4	7.37E-21	3.34E-21
Nuf2	0.1	1.3	4.28	3.08E-31	1.02E-31
Apol9a	0.1	1.1	4.25	3.14E-18	1.57E-18
Grem2	0.1	1.7	4.24	1.06E-66	1.80E-67
Gm2573	0.3	5.9	4.2	2.86E-49	6.31E-50
Ms4a4c	0.1	1.1	4.18	6.91E-21	3.13E-21
Ly6h	0.2	3.6	4.14	7.45E-39	2.04E-39
Gm266	0.1	1.4	4.05	6.28E-19	3.04E-19
Cxcl9	0.2	3.6	4.03	4.60E-106	5.02E-107
Hist1h2ap	1.6	22.9	3.88	7.95E-97	9.40E-98
Ccnd1	0.4	5.3	3.85	1.85E-201	1.06E-202
Apol9b	0.1	1.7	3.81	8.10E-27	3.03E-27
Mest	4	57	3.8	0	0
Frzb	0.5	6.3	3.75	5.89E-178	3.80E-179
Lst1	0.8	10.2	3.68	2.92E-39	7.93E-40
Mki67	0.1	1.4	3.63	4.06E-122	3.81E-123
Ptn	1.3	14.9	3.56	2.17E-186	1.33E-187
Ifit3b	0.3	3.2	3.55	3.65E-60	6.75E-61
Oas1a	0.3	3.6	3.5	2.68E-55	5.33E-56
Ifit3	0.8	9.2	3.47	6.39E-165	4.48E-166
Ccl12	0.1	1.3	3.46	9.97E-08	8.84E-08
Actc1	207.5	2247	3.45	0	0
Top2a	0.2	1.9	3.36	5.39E-88	7.09E-89
Col15a1	2.7	26.8	3.35	0	0
Oas2	0.3	3	3.27	7.71E-102	8.67E-103
Map2k6	1.1	10	3.25	7.13E-214	3.77E-215
Aplnr	1.8	16.7	3.23	0	0
Phf11d	0.2	2.2	3.23	1.59E-52	3.32E-53
Ifi44	0.2	2.2	3.2	1.01E-54	2.03E-55
Cdk1	0.2	1.5	3.17	1.45E-37	4.09E-38
Ccna2	0.4	3.5	3.16	4.08E-85	5.55E-86

Oasl2	1.4	12.4	3.12	1.30E-219	6.71E-221
Chga	0.2	1.6	3.11	1.06E-26	3.99E-27
Mybphl	0.3	2.4	3.08	5.75E-26	2.20E-26
Fcrls	0.4	3.2	3.06	2.15E-53	4.42E-54
Cntnap2	0.1	1.1	3.06	5.14E-31	1.72E-31
Ifit1	0.5	3.7	3.04	3.33E-81	4.66E-82
Th	0.2	1.3	3.04	1.83E-19	8.73E-20
Xlr3b	0.5	4.3	3.02	2.48E-55	4.92E-56
Cxcl10	0.4	3.5	3.02	1.10E-32	3.48E-33
Tbxa2r	0.1	1	3.02	2.45E-16	1.33E-16
Aif1	0.7	6	3.01	5.02E-43	1.25E-43
Isg15	1.1	8.5	2.96	1.24E-50	2.66E-51
Hist1h3e	0.1	1.1	2.92	4.55E-05	5.11E-05
Zbp1	0.3	2.5	2.9	9.35E-28	3.41E-28
Dctd	0.2	1.6	2.9	2.87E-24	1.16E-24
Cd72	0.3	2.3	2.89	2.77E-24	1.12E-24
Ccr5	0.2	1.1	2.89	4.90E-27	1.82E-27
Hdhd3	0.3	2.4	2.86	6.56E-21	2.97E-21
Mmp12	0.7	5.3	2.85	7.47E-75	1.14E-75
Fam64a	0.3	1.6	2.85	1.37E-19	6.51E-20
Psmb9	1.2	8.2	2.83	3.02E-51	6.42E-52
Itgax	0.5	3.2	2.83	6.80E-100	7.83E-101
Meox1	0.6	4.4	2.82	2.32E-75	3.53E-76
Ky	5.3	38.2	2.81	0	0
Usp18	0.4	2.9	2.81	1.07E-39	2.87E-40
Ifi27l2a	3.8	25.8	2.79	2.62E-85	3.54E-86
Ifit2	0.3	2.3	2.79	1.19E-68	1.97E-69
Ostn	0.2	1.3	2.79	3.15E-13	1.98E-13
A830010M20Rik	0.5	3.2	2.78	7.11E-159	5.20E-160
Ms4a4b	0.2	1.2	2.78	4.59E-13	2.92E-13
Bst2	5.8	39.2	2.77	9.29E-247	4.32E-248
Pcdh12	0.3	1.8	2.77	1.59E-76	2.36E-77
Fbxl22	1.5	9.8	2.76	1.70E-286	7.32E-288
Sox8	0.2	1.6	2.76	9.95E-37	2.87E-37
Irf7	2.3	14.8	2.73	4.20E-199	2.43E-200
Tceal7	55.3	356.6	2.71	0	0
Phf11b	0.5	3	2.71	1.52E-28	5.45E-29
H2-Ab1	10.9	69.5	2.7	0	0
Cx3cr1	0.3	1.6	2.7	2.06E-46	4.78E-47
Dlk1	6.5	41.4	2.69	0	0
Iigp1	1	6.8	2.67	4.04E-144	3.25E-145
H2-Q9	0.9	5.7	2.67	3.96E-63	7.08E-64
Kif23	0.2	1	2.67	7.07E-25	2.79E-25
Sdsl	0.2	1.4	2.66	1.59E-13	9.88E-14
Map3k7cl	6.4	39.6	2.65	0	0
Slfn1	0.2	1	2.63	5.40E-15	3.12E-15
Tnc	1.2	7.1	2.6	2.90E-298	1.20E-299
Socs1	0.6	3.4	2.6	4.57E-30	1.56E-30
Cryba4	0.2	1.1	2.59	1.71E-07	1.55E-07

Knstrn	0.2	1	2.57	2.53E-18	1.26E-18
Slamf9	0.4	2.4	2.56	8.53E-20	4.01E-20
Nos2	0.3	1.7	2.56	1.27E-44	3.07E-45
Tceal3	1	5.8	2.54	2.01E-43	4.98E-44
Fam26f	0.2	1	2.53	9.61E-09	7.95E-09
Cdkn2a	0.2	1.1	2.52	6.87E-08	6.02E-08
Fscn1	6.9	39.1	2.51	0	0
H2-Eb1	5.3	30	2.51	0	0
Ccl21a	1.8	10.3	2.5	4.12E-59	7.74E-60
Ptx3	1.7	9.5	2.5	1.91E-121	1.81E-122
Oasl1	0.4	2	2.49	7.93E-30	2.73E-30
Cyfip2	3.2	17.5	2.48	0	0
Nabp1	1.8	10.5	2.47	1.86E-171	1.27E-172
Gbp2	1.2	6.7	2.47	6.44E-109	6.89E-110
Ccl8	2.3	12.6	2.46	2.40E-41	6.24E-42
Gbp3	0.7	4	2.46	7.00E-70	1.15E-70
Nrep	8.3	45.1	2.45	0	0
Ccdc69	2.3	12.7	2.45	1.85E-117	1.80E-118
Tgtp2	0.6	3.1	2.42	1.24E-57	2.38E-58
Cdc20	0.2	1.3	2.42	3.04E-16	1.66E-16
Cdca8	0.3	1.6	2.41	1.71E-17	8.82E-18
Rrad	3.7	19.1	2.4	1.87E-191	1.12E-192
Spon2	0.5	2.6	2.4	1.82E-34	5.50E-35
2810417H13Rik	0.4	2.1	2.4	5.54E-33	1.75E-33
Fcgr1	0.4	2.2	2.39	5.63E-37	1.62E-37
Rasl10a	0.2	1.2	2.39	1.01E-09	7.78E-10
Xaf1	1	4.6	2.38	2.39E-53	4.93E-54
Hamp2	0.9	4.5	2.37	7.43E-13	4.78E-13
1810041L15Rik	0.6	3	2.37	7.77E-112	8.00E-113
Mdk	4.6	23.9	2.36	1.62E-115	1.61E-116
Tgtp1	0.4	1.8	2.36	3.52E-32	1.13E-32
Fam83d	0.3	1.3	2.36	1.31E-16	7.01E-17
Isoc2b	0.7	3.8	2.35	2.25E-25	8.75E-26
Gxylt2	0.2	1	2.35	6.90E-14	4.21E-14
Rtp4	1.5	7.7	2.34	1.62E-76	2.41E-77
Glipr2	1.1	5.3	2.34	1.26E-67	2.12E-68
1700040L02Rik	0.2	1.2	2.34	1.55E-10	1.14E-10
Serpinh1	17.9	89.3	2.33	0	0
Gatm	2.4	12	2.33	2.41E-174	1.60E-175
Aqp4	1.2	5.9	2.33	1.41E-183	8.86E-185
Ifi47	0.6	2.9	2.33	1.57E-32	5.00E-33
Bcl6b	1.2	5.7	2.3	1.11E-118	1.08E-119
Fam198b	0.9	4.5	2.3	2.64E-181	1.67E-182
Marcksl1	2.2	10.7	2.28	1.32E-103	1.47E-104
Batf3	0.2	1	2.28	1.08E-05	1.13E-05
H2-DMb1	1.3	6.1	2.27	1.33E-47	3.05E-48
Rsph1	0.7	3.6	2.26	1.73E-25	6.72E-26
Zmynd15	0.4	1.8	2.26	1.28E-23	5.33E-24
Gbp5	0.3	1.6	2.25	3.31E-29	1.16E-29

Prim1	0.2	1.1	2.25	2.49E-11	1.74E-11
Rcn3	6.6	30.7	2.24	5.60E-257	2.56E-258
Clec11a	1	4.4	2.22	5.15E-51	1.10E-51
Peg10	0.4	1.9	2.22	1.36E-75	2.05E-76
Sct	0.3	1.2	2.22	7.65E-05	8.77E-05
Slfn8	0.2	1.1	2.22	4.06E-23	1.71E-23
Krt222	0.2	1.1	2.21	4.03E-19	1.94E-19
Stmn1	3.2	14.7	2.2	6.37E-90	8.16E-91
Fkbp11	0.7	3.1	2.2	4.72E-15	2.72E-15
Kif22	0.3	1.6	2.2	1.89E-20	8.69E-21
Tmem14a	0.4	1.6	2.2	1.00E-11	6.83E-12
Cdkn2b	0.3	1.3	2.2	1.00E-11	6.83E-12
Sez6l2	0.3	1.2	2.2	6.29E-28	2.28E-28
Plagl1	4.2	18.7	2.19	0	0
Ccnd2	0.6	2.9	2.19	1.49E-101	1.68E-102
Apol10b	0.4	1.7	2.19	2.67E-21	1.19E-21
H2-Aa	17.3	77.3	2.18	0	0
Tnfaip8l1	0.3	1.5	2.18	4.60E-21	2.07E-21
Rec8	1.4	6.5	2.17	1.83E-69	3.00E-70
C1qtnf3	3.1	13.8	2.16	6.75E-184	4.21E-185
Kcnh2	0.4	1.9	2.16	8.66E-36	2.55E-36
Hdac9	0.3	1.6	2.16	2.78E-42	7.08E-43
Col8a2	0.2	1.1	2.16	1.07E-27	3.91E-28
Mmp23	1	4.1	2.15	6.48E-34	1.99E-34
Arsi	0.5	1.8	2.15	2.13E-32	6.84E-33
Rtkn2	0.2	1.3	2.15	5.72E-21	2.58E-21
Cenpa	1.1	4.3	2.14	5.20E-31	1.74E-31
Lsmem1	4.5	19.7	2.13	6.80E-129	6.13E-130
Cd4	0.3	1.2	2.13	1.04E-22	4.46E-23
Cx3cl1	1	4.1	2.12	2.73E-71	4.38E-72
Fam212b	0.9	3.9	2.12	7.47E-98	8.73E-99
Mov10	0.5	2.2	2.12	7.25E-44	1.78E-44
Tap1	1.1	4.8	2.11	8.01E-78	1.17E-78
Psmb8	2.1	9.1	2.1	2.86E-60	5.29E-61
Armcx6	0.6	2.5	2.1	3.90E-29	1.37E-29
Mad2l1	0.5	1.9	2.1	1.48E-18	7.33E-19
Racgap1	0.5	2.1	2.09	3.22E-34	9.80E-35
Nusap1	0.3	1	2.09	1.26E-14	7.42E-15
Dupd1	13.5	56.7	2.08	0	0
Pnck	2.6	11	2.08	3.63E-89	4.70E-90
Prr29	2.7	11.3	2.07	1.56E-45	3.70E-46
Tmem255b	0.9	3.9	2.07	2.60E-28	9.34E-29
Gm4070	0.3	1.2	2.07	4.88E-59	9.20E-60
Marcks	5.2	21.6	2.06	0	0
Parp10	0.8	3.5	2.06	5.97E-62	1.08E-62
Smco4	2.7	10.9	2.05	4.77E-59	8.98E-60
Pcp4l1	1.9	8.1	2.05	2.52E-66	4.31E-67
Uba7	0.7	2.7	2.05	3.67E-46	8.53E-47
Gbp7	0.6	2.5	2.05	1.43E-74	2.19E-75

Hddc3	3.4	23.3	2.04	7.37E-127	6.71E-128
Lxn	1.4	5.7	2.04	4.27E-33	1.34E-33
Hebp1	2.1	8.4	2.03	2.14E-46	4.96E-47
H2-Q6	0.9	3.8	2.03	1.97E-20	9.10E-21
Dok5	0.4	1.6	2.03	1.02E-15	5.72E-16
Apoa2	0.2	1	2.03	0.000356004	0.00044134
Crybb1	1.4	5.6	2.02	7.64E-26	2.93E-26
Nkg7	0.4	1.7	2.02	2.12E-08	1.80E-08
Ephx4	0.3	1.2	2.02	2.12E-08	1.80E-08
Rgs16	0.5	1.9	2.01	8.86E-24	3.65E-24
Il2rg	2	8	2	1.26E-66	2.15E-67
Dpysl3	1.3	5.3	2	5.58E-145	4.46E-146
Gm1673	0.5	1.9	2	1.60E-06	1.56E-06
Cd74	44.8	173.4	1.99	0	0
Itga9	1.1	4.4	1.99	2.01E-120	1.93E-121
Exoc3l4	0.5	2	1.99	3.80E-33	1.19E-33
Cmpk2	0.5	1.9	1.99	1.06E-31	3.47E-32
Nmrk2	34	156.8	1.98	0	0
Ndn	1.7	6.6	1.98	3.13E-55	6.24E-56
Ifi203	2.1	8.1	1.96	7.47E-160	5.41E-161
Stat1	1.7	6.9	1.96	8.54E-144	6.91E-145
Emilin1	1.3	5	1.96	3.48E-86	4.65E-87
Pik3ap1	0.4	1.5	1.96	4.08E-31	1.36E-31
Prelid2	0.5	1.8	1.95	8.62E-08	7.60E-08
Cd52	7.2	27.4	1.94	2.63E-68	4.40E-69
Nfkbie	0.3	1.2	1.94	3.85E-16	2.11E-16
Cstad	0.3	1.2	1.94	4.04E-06	4.10E-06
Tmem202	0.3	1.1	1.94	1.54E-06	1.51E-06
Trim21	0.3	1.1	1.94	1.81E-16	9.76E-17
Bex1	23.1	86.3	1.93	0	0
Chid1	1.2	5.3	1.93	1.00E-59	1.86E-60
Cd83	0.7	2.6	1.93	1.84E-28	6.61E-29
Tlr1	0.3	1.1	1.93	1.32E-16	7.08E-17
Armcx2	1.2	4.4	1.92	3.02E-74	4.65E-75
Crym	0.7	2.7	1.92	1.27E-17	6.51E-18
Gamt	28	104.5	1.91	0	0
Igtp	2.5	9.4	1.91	1.34E-92	1.68E-93
Ptgr1	1.4	5.2	1.91	2.37E-34	7.20E-35
Fgd2	0.6	2.2	1.91	1.89E-22	8.13E-23
Epsti1	0.6	2.2	1.91	3.72E-20	1.73E-20
Myl3	11.3	42.3	1.9	8.37E-174	5.58E-175
Dpt	7	25.7	1.9	3.75E-199	2.16E-200
Gimap4	2.3	9.1	1.9	1.96E-66	3.35E-67
Rcn1	1.7	6.4	1.9	1.06E-94	1.30E-95
Zfp365	1.1	4.1	1.9	5.60E-86	7.51E-87
2610042L04Rik	0.5	1.4	1.9	3.28E-12	2.18E-12
Mfap4	12.4	45.3	1.89	0	0
Col14a1	1.6	6	1.89	8.89E-184	5.57E-185
Dok4	1	3.6	1.88	6.45E-45	1.55E-45

Rpl34-ps1	25.8	93.6	1.87	1.48E-201	8.46E-203
Krt18	16.6	59.8	1.87	0	0
Ripk3	0.4	1.6	1.87	3.49E-15	2.00E-15
Cdkn1c	24.4	87.5	1.86	0	0
Tgfb1i1	4.7	17.8	1.86	5.41E-147	4.25E-148
Pcbd1	2.8	10.1	1.86	1.58E-38	4.38E-39
C1qtnf2	1.1	3.8	1.86	3.91E-22	1.70E-22
Fam171b	0.6	2.1	1.86	2.99E-57	5.78E-58
Fam57a	0.6	2	1.86	2.65E-17	1.38E-17
Sox11	0.4	1.2	1.86	2.65E-50	5.77E-51
Cited4	21.1	75.8	1.85	0	0
Cnn2	3.4	12	1.85	1.22E-99	1.41E-100
Kdr	1.8	6.3	1.85	3.41E-159	2.49E-160
S1pr3	0.6	2	1.85	3.59E-42	9.17E-43
Tnni3	0.3	1.1	1.85	6.04E-06	6.21E-06
Pcdh18	0.3	1.1	1.85	5.07E-27	1.89E-27
Gmppb	1.3	4.6	1.84	6.45E-38	1.80E-38
Serpinb9	0.8	2.8	1.84	3.39E-38	9.44E-39
Hba-a2	18.4	66	1.83	2.70E-164	1.90E-165
Fam71e1	2.5	7.9	1.83	2.85E-121	2.70E-122
Clec12a	0.9	3.2	1.83	9.31E-21	4.24E-21
Dhx58	0.6	1.9	1.83	1.24E-22	5.31E-23
Armcx1	0.5	1.9	1.83	1.55E-20	7.13E-21
Themis2	0.4	1.6	1.83	1.73E-27	6.33E-28
Lrrc15	0.4	1.5	1.83	9.16E-37	2.64E-37
Kctd12b	0.5	1.6	1.82	1.36E-36	3.96E-37
1700007K13Rik	0.4	1.3	1.82	2.16E-06	2.13E-06
Ush1c	0.3	1.2	1.82	3.21E-14	1.93E-14
Myog	15.8	54.5	1.81	0	0
Fam101b	4.4	15.2	1.81	1.52E-238	7.39E-240
Lrp2bp	1.5	5.3	1.81	7.00E-77	1.03E-77
Cks2	1	3.5	1.81	1.02E-11	6.99E-12
Fam19a3	0.7	2.5	1.81	1.60E-50	3.46E-51
Unc5b	0.5	1.6	1.81	9.83E-43	2.48E-43
Wisp1	0.3	1.1	1.81	5.92E-27	2.21E-27
Iqsec3	0.3	1	1.81	6.13E-32	1.99E-32
Mndal	3	11.2	1.8	1.00E-101	1.13E-102
Slc16a6	1	3.6	1.8	5.37E-66	9.21E-67
Tspan18	0.5	1.6	1.8	9.46E-28	3.45E-28
Bok	1.2	4.2	1.79	8.21E-30	2.83E-30
Cyp4f39	1.1	3.7	1.79	7.03E-42	1.81E-42
Tnfrsf19	1	3.3	1.79	8.87E-60	1.65E-60
Pyhin1	0.3	1	1.79	8.02E-17	4.25E-17
Lgals9	4.7	15.9	1.78	2.34E-103	2.61E-104
Ms4a7	3.3	11.2	1.78	3.38E-65	5.85E-66
Icam2	2.8	9.6	1.78	8.83E-49	1.97E-49
Ptprs	2.6	8.9	1.78	5.35E-200	3.07E-201
Dut	1	3.3	1.78	2.73E-34	8.29E-35
Lhfpl2	0.9	2.9	1.78	4.79E-57	9.29E-58

Igfbp2	0.8	2.8	1.78	5.94E-15	3.44E-15
Fcgr4	0.7	2.5	1.78	9.91E-15	5.82E-15
Parp14	0.7	2.4	1.78	2.37E-77	3.49E-78
Slc15a3	0.7	2.2	1.78	6.11E-24	2.51E-24
Col1a1	113.4	382.9	1.77	0	0
Cd247	0.8	2.2	1.77	1.89E-18	9.35E-19
2310033P09Rik	1.9	6.4	1.76	1.28E-34	3.86E-35
Kif11	0.4	1.3	1.76	4.00E-29	1.41E-29
Acta2	15.5	51.1	1.75	0	0
1500015O10Rik	0.5	1.8	1.75	5.79E-08	5.05E-08
Rnf213	0.5	1.7	1.75	3.16E-121	3.00E-122
Tuba1a	19.1	62.8	1.74	0	0
Gm13889	11.1	36.5	1.74	2.31E-197	1.36E-198
Ets1	1.8	6.2	1.74	1.15E-126	1.04E-127
Lurap1l	0.9	3.1	1.74	5.16E-23	2.18E-23
Ska2	0.5	1.8	1.74	1.25E-11	8.61E-12
P4ha3	0.5	1.6	1.74	5.27E-17	2.77E-17
Mcm5	0.4	1.4	1.74	3.47E-21	1.56E-21
Col24a1	0.4	1.3	1.74	3.40E-41	8.90E-42
Jchain	0.3	1	1.74	3.69E-10	2.77E-10
Crtap	4.5	14.9	1.73	5.47E-107	5.94E-108
Plxnd1	3.7	12.3	1.73	0	0
Basp1	1.6	5.3	1.73	5.53E-42	1.42E-42
Vmn1r65	0.9	2.9	1.73	8.11E-33	2.57E-33
Itgb3bp	0.6	2	1.73	2.28E-16	1.23E-16
Pycard	0.5	1.7	1.73	2.28E-16	1.23E-16
Ssu2	0.4	1.5	1.73	2.33E-10	1.73E-10
Ckb	3.9	12.7	1.72	1.48E-77	2.17E-78
Gm4841	3.7	12.2	1.72	1.31E-140	1.09E-141
Cxx1c	2.6	8.5	1.72	3.53E-34	1.08E-34
Gpr162	0.4	1.3	1.72	2.89E-17	1.50E-17
Adam12	0.4	1.3	1.72	1.34E-43	3.31E-44
Tm4sf1	3.5	11.3	1.71	1.26E-72	1.99E-73
Eps8l1	0.5	1.6	1.71	4.97E-30	1.70E-30
Serpinb6b	0.3	1	1.71	1.00E-08	8.30E-09
Gsta4	4	13.2	1.7	2.40E-51	5.07E-52
Kdelr3	2.3	7.5	1.7	5.52E-44	1.35E-44
Ctso	0.6	2	1.7	1.70E-29	5.89E-30
Ptx4	0.4	1.4	1.7	2.85E-08	2.44E-08
Sybu	0.3	1	1.7	1.59E-11	1.10E-11
Hba-a1	35.6	115	1.69	1.01E-246	4.70E-248
Col6a3	5.3	16.8	1.69	0	0
Lcp1	2.4	7.8	1.69	1.71E-111	1.77E-112
Dynll1	12.8	40.7	1.68	0	0
Lgals3bp	7.9	25.1	1.68	3.68E-217	1.91E-218
Mgl2	2	6.5	1.68	2.78E-39	7.56E-40
Angpt1	0.8	2.5	1.68	8.82E-39	2.43E-39
Exo5	0.4	1.2	1.68	1.04E-10	7.54E-11
Spats2l	1	2.7	1.67	9.87E-39	2.72E-39

Rwdd3	0.6	2	1.67	6.39E-13	4.09E-13
Zfp296	0.4	1.2	1.67	1.53E-08	1.29E-08
Dbp	18.9	60.6	1.66	0	0
Ift122	3.2	9.9	1.66	2.44E-160	1.76E-161
Mlkl	0.5	1.6	1.66	2.04E-13	1.27E-13
Ngfrap1	2.7	8.5	1.65	1.70E-31	5.59E-32
Col18a1	2.3	7.1	1.65	1.07E-142	8.80E-144
Nudt1	1.8	5.5	1.64	5.35E-21	2.41E-21
Rmi2	0.6	1.7	1.64	5.55E-26	2.12E-26
Lcp2	0.4	1.1	1.64	3.99E-16	2.19E-16
Lama4	1.5	4.7	1.63	5.86E-112	6.02E-113
Efs	0.6	1.8	1.63	9.65E-26	3.72E-26
Exoc3l2	0.3	1	1.63	2.67E-17	1.38E-17
Pcbd2	5.2	15.8	1.62	2.31E-35	6.86E-36
Ebpl	1.2	3.7	1.62	1.72E-30	5.84E-31
Prkar2b	0.4	1	1.62	8.74E-14	5.36E-14
Cd1d1	0.3	1	1.62	1.87E-08	1.58E-08
Myct1	1.9	5.9	1.61	2.12E-63	3.77E-64
Opn3	0.8	2.3	1.61	1.64E-14	9.70E-15
Vstm4	0.7	2.1	1.61	2.53E-22	1.10E-22
Hmgn2	30.6	92.2	1.6	0	0
Ckap4	6.3	18.7	1.6	1.87E-204	1.05E-205
Ccl5	2.9	8.8	1.6	1.76E-19	8.38E-20
Gm13139	0.5	1.6	1.6	6.37E-18	3.23E-18
LOC105247253	0.5	1.6	1.6	0.000420774	0.000526749
Rnf208	0.6	1.6	1.6	1.23E-09	9.56E-10
Vkorc1	8	24	1.59	6.80E-67	1.15E-67
Echdc3	1.3	4	1.59	6.00E-28	2.18E-28
Nrros	1.1	3.5	1.59	7.30E-33	2.31E-33
Zfp612	1.1	3.2	1.59	1.65E-58	3.13E-59
Cryl1	0.9	2.6	1.59	1.11E-14	6.52E-15
Alyref2	0.8	2.5	1.59	5.00E-13	3.19E-13
Fam111a	0.7	2.1	1.59	4.36E-26	1.66E-26
Tspan2	0.7	2.1	1.59	4.27E-33	1.34E-33
Tnfsf10	0.6	1.8	1.59	1.72E-34	5.21E-35
Mmp15	3.7	11	1.58	5.52E-175	3.63E-176
Tubb2b	2.4	7	1.58	2.33E-50	5.07E-51
Sepn1	1.6	4.7	1.58	2.61E-61	4.76E-62
Faim	1	3	1.58	1.88E-12	1.23E-12
Arhgap4	0.5	1.6	1.58	1.61E-16	8.64E-17
D1Ertd622e	1.4	4.2	1.57	1.51E-41	3.91E-42
Tnfaip3	1	3	1.57	1.36E-48	3.03E-49
Hck	0.4	1.3	1.57	1.50E-11	1.04E-11
Igfbp5	30.2	89	1.56	0	0
Col6a2	21.6	62.9	1.56	0	0
Pea15a	7.1	20.9	1.56	4.12E-187	2.51E-188
Gbp6	1.5	4.3	1.56	8.99E-67	1.53E-67
Bace2	0.3	1	1.56	6.48E-14	3.95E-14
Gdf1	5.6	17.1	1.55	2.87E-117	2.80E-118

Asb10	5.6	16.5	1.55	3.12E-134	2.70E-135
Col16a1	3.2	9.7	1.55	9.28E-130	8.32E-131
Pxdn	2.7	8	1.55	1.07E-151	8.14E-153
Tmtc1	2.2	6.5	1.55	4.66E-193	2.78E-194
H2-Q7	1.9	5.7	1.55	3.37E-25	1.32E-25
Jade1	1.2	3.6	1.55	1.61E-71	2.57E-72
Fhl2	1.2	3.4	1.55	2.59E-18	1.29E-18
Slc7a5	0.9	2.5	1.55	4.81E-33	1.51E-33
Cited1	0.8	2.4	1.55	3.31E-08	2.84E-08
Lag3	0.5	1.5	1.55	5.18E-12	3.48E-12
Lair1	0.4	1.1	1.55	2.02E-14	1.20E-14
Snx20	0.4	1.1	1.55	9.45E-08	8.36E-08
Rasgef1b	0.4	1	1.55	1.30E-12	8.47E-13
Ly6e	36.1	104.1	1.54	0	0
Ptges3l	27.3	79.9	1.54	6.24E-247	2.89E-248
Lmnb1	1.3	3.8	1.54	9.42E-40	2.53E-40
Nid2	0.9	2.6	1.54	6.66E-47	1.53E-47
Trmt12	0.7	2.1	1.54	1.43E-31	4.69E-32
B3galt6	0.4	1.2	1.54	1.52E-15	8.57E-16
Pfn4	0.4	1.1	1.54	2.25E-06	2.23E-06
Tmsb4x	179.5	519.1	1.53	0	0
Tnmd	3.9	11.2	1.53	1.29E-52	2.68E-53
Paqr7	2.6	8.5	1.53	3.11E-73	4.85E-74
Oaf	2.6	7.5	1.53	1.39E-63	2.46E-64
Tpst1	1.9	5.5	1.53	1.95E-37	5.54E-38
Gsdmd	1.8	5.1	1.53	5.67E-33	1.79E-33
Calml4	1.6	4.5	1.53	1.11E-13	6.86E-14
Sp110	1.2	3.3	1.53	1.31E-23	5.42E-24
Sox7	1.1	3.2	1.53	1.01E-37	2.83E-38
Esr1	0.9	2.6	1.53	1.01E-55	1.99E-56
Rtp3	0.5	1.5	1.53	2.65E-14	1.59E-14
Chst7	0.4	1	1.53	1.90E-09	1.50E-09
Fabp5	9.1	25.8	1.52	9.73E-86	1.31E-86
Gpx8	6	16.1	1.52	1.77E-54	3.58E-55
Capn6	3.2	9.1	1.52	3.03E-115	3.02E-116
Adar	0.7	2.1	1.52	6.70E-43	1.68E-43
Aurka	0.7	1.9	1.52	1.29E-13	8.00E-14
Car3	394.8	1132.5	1.51	0	0
Myl2	6.5	18.3	1.51	5.12E-40	1.36E-40
Thy1	5.4	15.1	1.51	1.67E-91	2.12E-92
Ogn	5.3	14.8	1.51	2.80E-129	2.51E-130
Ube2l6	3.8	10.6	1.51	1.80E-51	3.80E-52
Hic1	1.8	5	1.51	5.04E-54	1.03E-54
Asb18	0.8	2.2	1.51	2.93E-15	1.68E-15
Foxs1	0.7	1.9	1.51	5.19E-10	3.94E-10
Traf1	0.5	1.5	1.51	2.46E-14	1.47E-14
Pydc4	0.6	1.3	1.51	4.52E-10	3.41E-10
Zfp433	0.4	1.2	1.51	9.60E-12	6.56E-12
Ampd1	114.4	342.6	1.5	0	0

Eml1	4.8	13.6	1.5	7.05E-177	4.56E-178
Cpq	2.3	6.6	1.5	9.52E-44	2.34E-44
Nmi	1.3	3.7	1.5	6.55E-18	3.32E-18
Klhl23	1.1	3.2	1.5	1.42E-45	3.35E-46
Tcf19	1	2.9	1.5	3.13E-17	1.63E-17
Golga7b	0.8	2.4	1.5	3.68E-24	1.50E-24
B3gnt9	0.5	1.5	1.5	1.85E-13	1.15E-13
Rem1	0.5	1.3	1.5	1.98E-08	1.68E-08
Mppe1	1.7	4.9	1.49	8.17E-34	2.52E-34
Kera	1.7	4.7	1.49	2.76E-31	9.14E-32
Il15	1.3	3.3	1.49	2.18E-16	1.18E-16
Ephb3	0.6	1.7	1.49	6.85E-25	2.70E-25
Ebi3	0.4	1.3	1.49	1.37E-05	1.46E-05
Myadm	16.5	45.7	1.48	0	0
Cmss1	5.1	14.5	1.48	9.70E-53	2.01E-53
Fam114a1	2	5.5	1.48	4.37E-55	8.75E-56
Fxyd7	1.3	3.7	1.48	1.66E-09	1.30E-09
Alox12	0.5	1.6	1.48	1.34E-13	8.27E-14
Cd300ld	0.4	1.1	1.48	2.74E-10	2.04E-10
Igsf10	0.4	1	1.48	1.94E-29	6.76E-30
Tmsb10	203.3	559.5	1.47	0	0
Col1a2	78.3	213.2	1.47	0	0
Prkag3	34.6	95	1.47	0	0
Rhbdl1	9.3	26.9	1.47	1.01E-138	8.47E-140
Tspan9	5.3	14.7	1.47	1.87E-189	1.13E-190
Nagk	2.2	6.1	1.47	5.52E-26	2.10E-26
Cetn4	0.4	1.2	1.47	2.02E-05	2.19E-05
Pmaip1	0.4	1	1.47	2.69E-10	2.00E-10
Tubb6	15.2	41.2	1.46	1.26E-243	5.98E-245
Plcd4	4.7	13	1.46	6.25E-116	6.15E-117
C1qtnf6	4.5	12.4	1.46	1.20E-93	1.48E-94
Hbb-bt	1.5	4	1.46	1.67E-09	1.31E-09
Trim30a	0.7	2	1.46	2.14E-26	8.10E-27
Tpx2	0.7	1.9	1.46	1.43E-23	5.92E-24
Lrrc48	0.6	1.7	1.46	2.83E-12	1.87E-12
Tubb2a	6.6	17.9	1.45	1.28E-95	1.54E-96
Kctd17	6.3	17.5	1.45	2.38E-97	2.79E-98
Ddah2	4.5	12.2	1.45	8.12E-54	1.65E-54
Mdga1	3.6	8.5	1.45	1.15E-198	6.69E-200
Tgfb2	2	5.3	1.45	3.93E-86	5.26E-87
Gm13871	0.5	1.5	1.45	2.00E-07	1.81E-07
Zap70	0.5	1.2	1.45	1.85E-09	1.46E-09
Col5a1	7.2	19.4	1.44	0	0
Sp100	4	10.8	1.44	8.31E-96	1.00E-96
Efemp2	3.5	9.5	1.44	2.11E-48	4.71E-49
Coa4	3.3	8.7	1.44	3.10E-25	1.21E-25
Tceal1	2.3	6.2	1.44	2.21E-24	8.93E-25
Adam19	1.7	4.7	1.44	2.58E-81	3.60E-82
Plekha2	1.6	4.3	1.44	9.72E-72	1.55E-72

Fam212a	1.1	3.1	1.44	8.77E-12	5.97E-12
Tex38	1.1	3	1.44	4.97E-10	3.77E-10
Chek2	1.1	2.9	1.44	7.99E-18	4.07E-18
Gbp4	0.9	2.5	1.44	6.78E-37	1.96E-37
Cd59b	0.6	2.2	1.44	1.41E-10	1.03E-10
Wnt5b	0.6	1.5	1.44	5.23E-12	3.52E-12
Cd274	0.5	1.2	1.44	8.04E-16	4.47E-16
Tuba1b	26.6	70.7	1.43	0	0
Igf1	1.8	4.8	1.43	4.47E-109	4.77E-110
H2-DMa	1.4	4.4	1.43	8.31E-28	3.03E-28
Stat2	1.3	3.6	1.43	1.04E-49	2.29E-50
Hn1l	1	2.8	1.43	4.07E-24	1.66E-24
Ptpn6	0.9	2.4	1.43	1.44E-18	7.09E-19
Adipoq	0.8	2.1	1.43	2.35E-09	1.86E-09
Lix1	0.7	1.8	1.43	1.99E-18	9.88E-19
Tril	0.6	1.7	1.43	2.46E-30	8.38E-31
Hacd4	0.7	1.6	1.43	1.63E-16	8.77E-17
Hspa12a	0.5	1.3	1.43	5.71E-25	2.25E-25
Cxcl16	3.1	8.1	1.42	4.39E-62	7.92E-63
Chchd6	1.2	3.3	1.42	2.04E-12	1.34E-12
Ldoc1l	0.7	2	1.42	4.11E-29	1.45E-29
Hps1	0.7	1.8	1.42	3.17E-17	1.66E-17
Sfrp2	0.6	1.7	1.42	9.60E-12	6.56E-12
P2ry1	0.6	1.6	1.42	6.76E-20	3.17E-20
Wdr5b	0.6	1.5	1.42	3.76E-10	2.82E-10
Scube2	0.4	1	1.42	3.43E-12	2.28E-12
Islr	7.1	18.5	1.41	3.29E-121	3.13E-122
Grb14	6.1	16.5	1.41	1.38E-97	1.62E-98
Mthfsd	2.7	6.5	1.41	2.59E-35	7.70E-36
Dhcr7	2.2	5.7	1.41	5.23E-44	1.28E-44
Inpp5j	0.9	2.4	1.41	1.92E-27	7.04E-28
Mfsd7a	0.9	2.4	1.41	1.64E-19	7.81E-20
Pafah1b3	0.8	2.2	1.41	1.96E-07	1.78E-07
Nrp	0.4	1.1	1.41	2.37E-05	2.58E-05
Hoxb7	0.4	1.1	1.41	6.80E-06	7.03E-06
Cxcl12	8.5	23.5	1.4	1.93E-198	1.13E-199
Irf5	2.9	7.6	1.4	3.43E-49	7.58E-50
Limd2	2.8	7.4	1.4	1.53E-37	4.31E-38
Trim12c	0.9	2.4	1.4	2.82E-27	1.04E-27
Slc26a10	0.7	1.8	1.4	9.53E-14	5.85E-14
Gale	0.6	1.6	1.4	4.96E-09	4.01E-09
Neu2	7.7	20.2	1.39	1.65E-96	1.97E-97
Mthfd2	1.9	5	1.39	1.01E-32	3.20E-33
Cfap126	1.4	3.8	1.39	2.41E-12	1.59E-12
Tmem116	1.1	3	1.39	1.44E-12	9.40E-13
Snai2	0.9	2.4	1.39	1.89E-16	1.02E-16
Lpcat2	0.8	1.8	1.39	4.45E-13	2.83E-13
Actb	150.8	390.1	1.38	0	0
Cav1	38.9	102.9	1.38	0	0

Tspan13	19.4	50.1	1.38	5.07E-286	2.19E-287
Gng11	14.8	38.3	1.38	3.34E-95	4.06E-96
Smco1	14.6	38.2	1.38	5.59E-141	4.62E-142
Pde4a	8.7	23	1.38	3.57E-285	1.55E-286
Mrc2	2	5.1	1.38	4.34E-92	5.46E-93
Prc1	1.2	3	1.38	5.24E-27	1.95E-27
Sertad4	0.9	2.4	1.38	8.60E-25	3.41E-25
Gm40368	0.9	2.2	1.38	1.76E-14	1.05E-14
Hist1h3d	0.8	2.1	1.38	0.000217374	0.000262709
Spc25	0.7	1.8	1.38	6.20E-09	5.04E-09
Lbx2	0.7	1.7	1.38	1.66E-05	1.78E-05
Tpm4	12.7	32.7	1.37	1.61E-202	9.08E-204
Mt3	11.4	29.7	1.37	1.36E-45	3.22E-46
Akr1e1	4.6	11.8	1.37	1.20E-60	2.21E-61
Ube2c	3.6	9.2	1.37	3.67E-26	1.40E-26
Pdia4	2.5	6.6	1.37	4.79E-51	1.02E-51
Mgmt	2	5.3	1.37	2.37E-14	1.41E-14
Dclk1	1.5	3.8	1.37	1.09E-64	1.91E-65
E130311K13Rik	1	2.6	1.37	9.37E-14	5.75E-14
Lig1	0.6	1.5	1.37	5.59E-16	3.09E-16
Tmem45a	0.5	1.4	1.37	2.89E-08	2.47E-08
Parp9	2.4	6.1	1.36	1.30E-57	2.49E-58
Dpysl2	1.2	3	1.36	1.80E-40	4.77E-41
Slc46a1	0.7	1.8	1.36	1.39E-11	9.59E-12
H2-M3	0.7	1.7	1.36	1.28E-08	1.07E-08
Kif20a	0.4	1	1.36	5.87E-12	3.96E-12
Rbl1	0.4	1	1.36	7.18E-12	4.87E-12
Bin2	0.4	1	1.36	7.10E-07	6.75E-07
Col3a1	79.5	200.3	1.35	0	0
Krt8	9.6	24.2	1.35	2.56E-126	2.34E-127
Ifi35	7.4	18.5	1.35	1.34E-72	2.11E-73
Steap3	6.8	17.2	1.35	1.26E-137	1.06E-138
Hmgb2	4.6	11.9	1.35	4.54E-42	1.16E-42
Coro1a	2.3	5.7	1.35	5.17E-29	1.83E-29
Ggcx	1.6	4	1.35	1.52E-35	4.48E-36
Eme2	1.3	3.2	1.35	1.33E-15	7.51E-16
Cdca3	1.1	3	1.35	1.61E-14	9.55E-15
Sdf2l1	1.1	2.6	1.35	6.47E-10	4.94E-10
Pigb	0.8	2.1	1.35	3.92E-14	2.37E-14
Primpol	0.5	1.2	1.35	3.16E-15	1.81E-15
Adamts14	0.4	1.1	1.35	1.86E-17	9.64E-18
Gm684	0.4	1.1	1.35	1.23E-05	1.30E-05
Azin1	10.1	26.7	1.34	5.34E-295	2.23E-296
Lrrc51	4	10.3	1.34	1.93E-26	7.28E-27
Irf1	2.5	6.4	1.34	8.49E-40	2.28E-40
Rgs2	2.2	5.5	1.34	2.43E-48	5.45E-49
Tspan6	1.8	4.5	1.34	1.42E-23	5.92E-24
Col8a1	1.7	4.3	1.34	2.53E-64	4.44E-65
Ripply1	1.3	3.3	1.34	1.06E-07	9.41E-08

Il17d	0.9	2.2	1.34	2.53E-09	2.00E-09
Psmc3ip	0.6	1.6	1.34	1.06E-05	1.11E-05
Plekha7	0.5	1.4	1.34	7.35E-19	3.57E-19
Efna2	0.4	1.1	1.34	3.07E-08	2.63E-08
Mfap2	10.6	25.5	1.33	4.38E-76	6.57E-77
Gm7325	2.4	6	1.33	6.50E-15	3.77E-15
Fsd1l	1.5	3.9	1.33	6.11E-80	8.70E-81
Lmnb2	1.5	3.7	1.33	2.34E-37	6.68E-38
Osbpl6	1.2	3	1.33	2.33E-69	3.83E-70
Kbtbd3	1.1	2.8	1.33	8.28E-21	3.76E-21
Mcm3	1	2.6	1.33	2.23E-22	9.63E-23
Chad	0.9	2.4	1.33	2.50E-12	1.65E-12
Ncf4	0.9	2.1	1.33	3.11E-09	2.48E-09
Clec4n	0.9	2	1.33	2.26E-08	1.92E-08
Xxylt1	0.8	1.9	1.33	1.06E-16	5.66E-17
Uhrf1	0.5	1.3	1.33	1.52E-13	9.44E-14
B2m	135.3	333.8	1.32	0	0
Lbh	3.2	8	1.32	3.56E-70	5.78E-71
Creb3l1	2.6	6.4	1.32	3.74E-42	9.55E-43
Prmt2	1.8	4.5	1.32	1.59E-27	5.83E-28
Gm3435	0.9	2.2	1.32	1.72E-11	1.19E-11
Duoxa1	0.7	1.8	1.32	1.00E-08	8.29E-09
L3hypdh	0.5	1.3	1.32	1.43E-07	1.28E-07
Tsc22d4	23.7	58.4	1.31	0	0
Cotl1	6.8	16.7	1.31	8.36E-77	1.24E-77
Ctnna3	0.9	2.3	1.31	6.63E-20	3.11E-20
B4galt2	0.9	2.1	1.31	8.05E-16	4.48E-16
Endod1	0.8	1.9	1.31	6.52E-23	2.77E-23
1700016C15Rik	0.6	1.5	1.31	0.000189332	0.000227149
B4galt6	0.6	1.5	1.31	7.36E-26	2.82E-26
S1pr2	0.5	1.3	1.31	5.08E-12	3.41E-12
Odf3l1	0.5	1.1	1.31	9.67E-05	0.000112157
Angptl2	24	58.7	1.3	0	0
Cbr2	4.5	11.4	1.3	4.15E-29	1.46E-29
Lamb1	3.2	7.9	1.3	1.44E-123	1.33E-124
Rccd1	1.6	4.1	1.3	3.01E-27	1.11E-27
Col26a1	1.5	3.8	1.3	2.57E-29	8.97E-30
Ass1	1.4	3.5	1.3	6.00E-17	3.17E-17
D3Ertd751e	0.7	1.8	1.3	8.64E-15	5.05E-15
Cd109	0.6	1.4	1.3	1.35E-24	5.39E-25
Spry4	0.6	1.3	1.3	2.29E-18	1.14E-18
Igfbp4	21.7	52.5	1.29	1.94E-293	8.17E-295
Dbn1	3.9	9.5	1.29	4.13E-76	6.19E-77
Sulf2	3	7.2	1.29	4.59E-73	7.20E-74
Fbxw17	1.7	4	1.29	1.99E-20	9.17E-21
Gstt1	1.4	3.3	1.29	1.24E-10	9.01E-11
Prpsap2	1.2	2.8	1.29	1.22E-14	7.21E-15
Hps4	1	2.6	1.29	2.75E-22	1.19E-22
Creb5	0.6	1.5	1.29	1.37E-30	4.64E-31

Aldh1a2	0.5	1.3	1.29	3.95E-09	3.18E-09
E430018J23Rik	0.4	1.1	1.29	2.48E-07	2.27E-07
Stmn2	0.5	1.1	1.29	2.48E-07	2.26E-07
Sparc	129.2	310.5	1.28	0	0
LOC102634333	7.3	17.7	1.28	0	0
Atf3	4.8	11.5	1.28	3.05E-62	5.49E-63
Wfdc17	3.1	7.4	1.28	1.75E-10	1.29E-10
Nudt18	2.3	5.6	1.28	1.48E-27	5.41E-28
Tmem218	2.2	5.6	1.28	1.77E-16	9.53E-17
Commd10	1.9	4.6	1.28	6.61E-20	3.10E-20
Mmgt2	1.7	4.2	1.28	9.02E-19	4.40E-19
Itih5	1.5	3.6	1.28	4.43E-77	6.52E-78
Col12a1	1.2	3	1.28	7.63E-84	1.05E-84
Hist1h2be	1.3	1.8	1.28	1.13E-11	7.74E-12
Cntrob	0.7	1.8	1.28	4.07E-18	2.04E-18
Cdk5r1	0.6	1.5	1.28	9.02E-19	4.40E-19
Tmeff1	0.5	1.1	1.28	8.89E-09	7.31E-09
Dtymk	7.6	18.1	1.27	1.38E-39	3.72E-40
Ccdc80	5.2	12.2	1.27	3.46E-113	3.50E-114
Chtf8	4.7	11.3	1.27	2.23E-86	2.98E-87
Praf2	3.4	8.1	1.27	1.10E-26	4.15E-27
St3gal4	1.8	4.3	1.27	8.18E-70	1.34E-70
Aqp7	1.7	3.8	1.27	2.47E-25	9.64E-26
Slc2a12	0.8	1.9	1.27	2.17E-19	1.04E-19
Ica1	0.5	1.4	1.27	1.54E-07	1.39E-07
Begain	0.5	1.3	1.27	2.11E-10	1.56E-10
Plk1	0.6	1.3	1.27	3.45E-09	2.76E-09
Nrn1	0.5	1.3	1.27	5.81E-07	5.48E-07
Tifab	0.5	1.2	1.27	9.31E-11	6.75E-11
Dagla	0.5	1.2	1.27	1.85E-18	9.15E-19
S100a11	46.5	109.8	1.26	1.38E-135	1.18E-136
Psme1	24.6	58.7	1.26	9.24E-145	7.41E-146
Morn2	6.2	13.9	1.26	3.98E-24	1.62E-24
Fkbp10	4.5	10.5	1.26	1.70E-72	2.68E-73
Akr1b8	4	9.4	1.26	1.33E-32	4.24E-33
Neu3	1	2.5	1.26	3.41E-23	1.43E-23
Accs	0.5	1.2	1.26	2.61E-13	1.64E-13
Psmb10	7.2	17.2	1.25	1.94E-57	3.73E-58
Srek1ip1	4.4	10.3	1.25	1.17E-47	2.67E-48
Gng10	3.5	8.3	1.25	7.37E-25	2.92E-25
Cd44	2.9	6.9	1.25	6.17E-80	8.80E-81
LOC108167320	2.3	5.5	1.25	7.24E-10	5.54E-10
P3h3	2.2	5.1	1.25	6.38E-36	1.87E-36
Pgpep1l	1.4	3.2	1.25	6.88E-11	4.95E-11
Sema6a	1.3	3	1.25	5.23E-53	1.08E-53
Gm12942	0.9	2.1	1.25	1.58E-07	1.42E-07
Lyplal1	0.8	1.9	1.25	1.57E-08	1.32E-08
Prrg3	0.8	1.8	1.25	3.69E-29	1.29E-29
Nipa1	0.5	1.2	1.25	1.85E-07	1.68E-07

E2f1	0.5	1.2	1.25	1.30E-09	1.01E-09
Pcolce	19.9	46.9	1.24	8.75E-184	5.46E-185
Col6a1	19.6	45.6	1.24	0	0
Casp7	1	2.4	1.24	9.98E-16	5.59E-16
Gm3558	0.8	1.8	1.24	4.04E-11	2.87E-11
Fam84b	0.5	1.1	1.24	8.99E-17	4.78E-17
Cfl1	70.2	162.7	1.23	0	0
Igfbp7	62.2	145	1.23	0	0
Postn	45.3	103.3	1.23	0	0
Mpp1	5.2	12	1.23	7.48E-77	1.11E-77
Fbxw7	4.4	10.7	1.23	5.96E-103	6.67E-104
Chrng	2.5	5.7	1.23	3.01E-28	1.08E-28
Bhlhb9	1.4	3.2	1.23	5.67E-24	2.33E-24
Fzd10	0.8	1.8	1.23	8.59E-15	5.02E-15
Sp140	0.8	1.7	1.23	4.36E-10	3.29E-10
Ccdc8	0.7	1.6	1.23	1.16E-09	8.99E-10
Raver2	0.5	1.2	1.23	5.25E-13	3.36E-13
Myoz3	25	58.6	1.22	0	0
Dap	9.1	21.1	1.22	2.57E-77	3.77E-78
Tra2a	7.4	18.3	1.22	2.27E-92	2.85E-93
Ikbip	3.3	7.1	1.22	7.85E-31	2.64E-31
Alg2	2.7	6.3	1.22	4.26E-48	9.58E-49
Nme4	2.7	6.1	1.22	2.75E-14	1.65E-14
Adamts20	2.1	5.4	1.22	3.72E-88	4.88E-89
Tmem119	2	4.6	1.22	1.60E-25	6.19E-26
Mcm6	1.4	3.2	1.22	5.86E-26	2.24E-26
H2-T24	1	2.5	1.22	9.04E-13	5.85E-13
AW551984	1	2.3	1.22	7.99E-23	3.40E-23
Rbm43	0.8	1.8	1.22	1.38E-10	1.01E-10
Ifitm3	61.8	141.4	1.21	2.91E-214	1.53E-215
Ifi27	13.7	31.6	1.21	1.03E-78	1.49E-79
Lum	13.5	30.9	1.21	9.33E-157	6.92E-158
Nes	10.6	24.2	1.21	0	0
Txndc5	9.8	22.6	1.21	2.45E-147	1.91E-148
Fap	2.3	5.1	1.21	8.62E-31	2.90E-31
Arsb	1	2.4	1.21	8.05E-25	3.19E-25
Ptprcap	0.8	1.8	1.21	3.20E-05	3.53E-05
Nudt17	0.6	1.4	1.21	8.91E-06	9.29E-06
Clec3b	7.3	16.6	1.2	6.96E-41	1.83E-41
Cthrc1	5.3	11.8	1.2	3.41E-33	1.07E-33
Sh3kbp1	4.8	11.2	1.2	3.62E-96	4.33E-97
Gngt2	4	9.4	1.2	9.36E-15	5.48E-15
Cercam	2.4	5.8	1.2	5.69E-32	1.84E-32
Ccdc34	2.4	5.4	1.2	1.41E-28	5.05E-29
Faap100	2.1	4.9	1.2	1.12E-38	3.09E-39
Comtd1	1.7	3.8	1.2	6.86E-22	3.01E-22
Cfb	1.2	2.7	1.2	1.30E-19	6.15E-20
Msx1	1	2.2	1.2	8.61E-12	5.86E-12
Anxa2	23.9	53.6	1.19	8.04E-177	5.22E-178

Tmem8c	18	40.5	1.19	6.89E-127	6.26E-128
Sec61b	15.7	35.8	1.19	1.43E-45	3.38E-46
Ostc	14.6	33.1	1.19	5.68E-84	7.79E-85
Lsm3	14.3	32.3	1.19	2.80E-47	6.41E-48
Vwa1	4.4	10	1.19	1.21E-45	2.86E-46
Hrasls	4.1	9.4	1.19	8.42E-66	1.45E-66
Plau	2.7	6	1.19	1.77E-33	5.52E-34
Rpa3	2.6	5.8	1.19	3.74E-10	2.81E-10
Cdc42ep5	2.3	5.1	1.19	1.41E-11	9.73E-12
Tk1	2	4.5	1.19	2.65E-15	1.51E-15
Prss53	1	2.5	1.19	4.05E-17	2.12E-17
Egflam	1	2.4	1.19	4.20E-21	1.89E-21
Smim1	1	2	1.19	5.32E-15	3.08E-15
Sytl2	0.8	1.8	1.19	1.46E-15	8.27E-16
Fgd1	0.5	1.2	1.19	4.66E-12	3.12E-12
Olfml1	0.5	1.1	1.19	9.96E-07	9.57E-07
1700037H04Rik	4.6	10.2	1.18	6.38E-31	2.14E-31
Mettl11b	2.4	5.4	1.18	2.37E-14	1.41E-14
Hdac11	2.4	5.4	1.18	1.69E-29	5.86E-30
Ccdc163	2	4.7	1.18	3.31E-15	1.90E-15
Plod2	2	4.5	1.18	4.29E-40	1.14E-40
Emid1	1.6	3.6	1.18	5.49E-16	3.03E-16
Tmem106a	1.6	3.5	1.18	9.45E-21	4.31E-21
Ptpn18	1.5	3.4	1.18	2.35E-13	1.47E-13
Plac8	1.1	2.7	1.18	5.19E-06	5.30E-06
Nmral1	1	2.3	1.18	2.67E-09	2.12E-09
Epb41l4a	0.6	1.4	1.18	7.24E-12	4.92E-12
Cchcr1	0.6	1.3	1.18	8.47E-10	6.52E-10
Wbp5	18.7	41.8	1.17	4.95E-97	5.83E-98
Pisd	15.1	33.3	1.17	7.37E-174	4.91E-175
Selm	13.5	30.1	1.17	7.84E-61	1.44E-61
Rgs10	5.4	12	1.17	4.35E-25	1.71E-25
Metrn	5.2	11.7	1.17	4.77E-37	1.37E-37
Fam171a2	2.4	5.3	1.17	2.56E-39	6.94E-40
C330018D20Rik	1.8	4.1	1.17	1.11E-23	4.60E-24
Clec4a3	1.1	2.4	1.17	6.04E-08	5.28E-08
Iqcg	0.8	1.9	1.17	1.23E-18	6.05E-19
Trdmt1	0.6	1.3	1.17	4.31E-12	2.88E-12
Fbxo27	0.5	1.1	1.17	3.15E-06	3.16E-06
LOC102638183	6.3	13.9	1.16	3.43E-13	2.16E-13
4-Sep	6.2	13.7	1.16	5.75E-44	1.41E-44
Fkbp7	6	13.2	1.16	7.11E-28	2.58E-28
Arap1	3.5	8	1.16	7.82E-65	1.37E-65
Vcpkmt	3.1	6.8	1.16	7.26E-19	3.53E-19
Pf4	2.8	6.1	1.16	1.16E-09	9.03E-10
Dtx3l	1.7	3.8	1.16	8.65E-46	2.03E-46
Casp8	1.6	3.6	1.16	2.82E-21	1.26E-21
Golm1	1.4	3.2	1.16	3.66E-27	1.35E-27
Pik3cd	1.3	3.1	1.16	1.04E-31	3.41E-32

Tmem185b	1.1	2.4	1.16	4.62E-17	2.43E-17
Pdia5	1	2.3	1.16	7.17E-11	5.17E-11
Siae	0.8	1.9	1.16	6.38E-16	3.53E-16
Mboat1	0.7	1.4	1.16	4.35E-10	3.28E-10
Gpr65	0.6	1.4	1.16	2.63E-08	2.25E-08
Plekhn1	0.6	1.4	1.16	2.23E-08	1.89E-08
Ptma	70.6	154.8	1.15	0	0
Aig1	6.1	12.9	1.15	2.09E-46	4.84E-47
Supt3	5	12.1	1.15	9.83E-32	3.21E-32
Slfn5	3.3	7.4	1.15	1.88E-101	2.13E-102
Pycr1	2.6	6.2	1.15	3.54E-27	1.31E-27
Casp3	2	4.5	1.15	8.07E-26	3.10E-26
Mall	1.9	4.2	1.15	1.24E-19	5.90E-20
Parp12	1.2	2.7	1.15	1.27E-21	5.63E-22
Senp8	1.1	2.4	1.15	1.51E-21	6.68E-22
Grtp1	1	2.4	1.15	3.63E-09	2.92E-09
Tppp3	18.6	44.4	1.14	3.49E-110	3.66E-111
Ppic	14.3	31.2	1.14	1.79E-88	2.33E-89
Snrpd1	10.4	22.8	1.14	3.14E-42	8.01E-43
Ssc5d	4.9	10.6	1.14	3.03E-103	3.38E-104
Gpx7	4.8	10.5	1.14	7.69E-29	2.73E-29
Impa2	3.8	8.2	1.14	2.45E-26	9.29E-27
Lysmd2	3.5	7.8	1.14	1.46E-21	6.48E-22
Tmem173	2.4	5.1	1.14	9.21E-25	3.66E-25
Arhgef15	2.2	4.8	1.14	6.81E-45	1.63E-45
Ppm1f	2	4.3	1.14	2.39E-47	5.48E-48
Tvp23b	1.3	2.9	1.14	1.60E-12	1.05E-12
Podn	1.3	2.9	1.14	3.87E-17	2.03E-17
Ptrh1	1	2.2	1.14	6.70E-06	6.93E-06
Mcm4	1	2.1	1.14	2.76E-18	1.38E-18
Chsy1	0.8	1.8	1.14	6.28E-17	3.32E-17
Ints2	0.6	1.3	1.14	1.44E-18	7.11E-19
F2rl3	0.5	1.3	1.14	6.01E-07	5.67E-07
Spon1	0.6	1.3	1.14	1.99E-19	9.50E-20
Igsf3	0.6	1.2	1.14	3.26E-21	1.46E-21
Myl9	14.7	32	1.13	2.36E-76	3.51E-77
Zfp385b	5.4	11.8	1.13	5.17E-71	8.32E-72
2210016F16Rik	3.6	7.9	1.13	5.67E-50	1.24E-50
Tap2	2.9	6.3	1.13	1.21E-35	3.57E-36
Arhgap18	1.5	3.2	1.13	1.03E-26	3.87E-27
Dusp19	1.3	2.9	1.13	1.56E-10	1.15E-10
Zfp827	1.4	2.9	1.13	1.13E-52	2.35E-53
Rap2b	1.3	2.8	1.13	4.48E-27	1.66E-27
Slc22a17	1.2	2.7	1.13	5.87E-15	3.40E-15
3110009E18Rik	1.2	2.5	1.13	0.000140855	0.000166593
Hvcn1	0.9	2.1	1.13	3.37E-13	2.12E-13
Nup35	0.8	1.8	1.13	2.59E-12	1.71E-12
Tmem35	0.8	1.8	1.13	4.92E-09	3.97E-09
Ttc30b	0.7	1.6	1.13	1.34E-10	9.80E-11

Serpinb8	0.7	1.5	1.13	3.22E-13	2.03E-13
Cyp51	0.6	1.4	1.13	2.77E-14	1.66E-14
Cntnap1	0.5	1.2	1.13	6.94E-15	4.03E-15
Myo1g	0.5	1.1	1.13	2.17E-09	1.72E-09
Aimp2	63.8	142.6	1.12	0	0
C1qa	35.8	76.5	1.12	2.78E-175	1.82E-176
Snrpf	12.9	28	1.12	4.88E-52	1.03E-52
Serf1	9	19.3	1.12	8.52E-26	3.28E-26
Pop7	6.6	14.2	1.12	7.18E-25	2.84E-25
Sdc2	5.1	11	1.12	5.07E-80	7.21E-81
Copg2	3.4	7.3	1.12	4.97E-64	8.77E-65
Clec7a	2.7	5.9	1.12	1.18E-28	4.21E-29
Chst14	1.2	2.5	1.12	1.11E-12	7.19E-13
Lrrcc1	1.1	2.5	1.12	6.99E-28	2.54E-28
Pdzd7	1.1	2.4	1.12	1.15E-21	5.08E-22
Ctbs	0.7	1.6	1.12	4.14E-11	2.94E-11
Galnt5	0.7	1.5	1.12	1.82E-17	9.41E-18
Proser2	0.5	1.1	1.12	1.80E-09	1.41E-09
H2-T22	7.1	15.3	1.11	1.73E-55	3.42E-56
Hmgb3	3.9	8.8	1.11	4.23E-31	1.41E-31
P4ha1	3.8	8.2	1.11	3.68E-70	5.99E-71
Ybx2	3.2	7.6	1.11	7.53E-28	2.74E-28
Med19	3.3	7.1	1.11	2.23E-32	7.15E-33
Nme3	2.7	5.9	1.11	3.99E-10	3.00E-10
Fam3c	2.5	5.3	1.11	4.07E-32	1.31E-32
Slc25a1	1.7	3.7	1.11	1.50E-14	8.86E-15
Rps6ka1	1.2	2.5	1.11	1.09E-17	5.60E-18
Tmem44	1	2.3	1.11	2.55E-18	1.27E-18
Zfp13	0.7	1.6	1.11	1.28E-08	1.07E-08
Lpar4	0.5	1.1	1.11	1.27E-11	8.75E-12
Coa3	37.2	79.5	1.1	2.76E-115	2.75E-116
Psme2	15	33.3	1.1	8.16E-59	1.54E-59
Pak1	12.7	27.2	1.1	1.93E-175	1.26E-176
Sox18	10.4	22.1	1.1	2.88E-75	4.38E-76
Ehd2	10.3	21.9	1.1	4.01E-235	1.97E-236
Bphl	7.5	16	1.1	2.91E-41	7.59E-42
Gm572	5.9	12.7	1.1	4.71E-65	8.18E-66
Sdpr	5.8	12.3	1.1	1.24E-80	1.76E-81
Sec14l5	5.4	11.5	1.1	2.03E-143	1.65E-144
Tmem176a	5.1	11	1.1	2.32E-24	9.36E-25
Foxo6	4.1	8.9	1.1	3.86E-44	9.42E-45
Pigh	2.2	4.7	1.1	5.00E-25	1.97E-25
Cacnb3	1.9	4.1	1.1	5.30E-23	2.24E-23
Magix	1.7	3.8	1.1	4.36E-23	1.84E-23
Neurl3	1.7	3.6	1.1	3.85E-21	1.73E-21
Fam229b	1.7	3.6	1.1	7.80E-07	7.44E-07
F830016B08Rik	1.6	3.4	1.1	3.89E-26	1.48E-26
Cbx8	1.1	2	1.1	8.96E-09	7.38E-09
Epb41l3	0.9	1.9	1.1	3.55E-17	1.86E-17

Tcaf2	0.7	1.4	1.1	5.38E-14	3.26E-14
Srpk3	22.7	48.1	1.09	3.06E-198	1.79E-199
Cyba	19.8	41.8	1.09	5.12E-65	8.91E-66
Prkcdbp	15.5	32.7	1.09	1.76E-68	2.94E-69
Tceal8	11.1	23.5	1.09	6.68E-111	6.96E-112
Tnfaip2	10.5	22.2	1.09	2.24E-162	1.60E-163
Tmem88	5.7	12.1	1.09	1.19E-40	3.15E-41
Rnpep	5.2	11	1.09	6.01E-52	1.26E-52
Mina	4.5	9.6	1.09	1.18E-43	2.92E-44
Mmab	4.5	9.5	1.09	2.02E-59	3.78E-60
Plpp5	3.6	7.4	1.09	7.69E-24	3.17E-24
Ephx2	3	6.3	1.09	7.52E-28	2.73E-28
Fam43a	2.5	5.4	1.09	3.55E-35	1.06E-35
Ppp1r14a	1.3	2.8	1.09	0.000223737	0.000270942
Alg6	1.3	2.6	1.09	1.20E-14	7.06E-15
Elp6	1.1	2.3	1.09	3.80E-11	2.69E-11
Adam33	0.9	1.8	1.09	7.72E-17	4.09E-17
Samd9l	0.7	1.5	1.09	7.13E-18	3.62E-18
Mchr1	0.5	1.1	1.09	2.94E-06	2.94E-06
Ubxn11	0.5	1.1	1.09	5.89E-05	6.67E-05
Tubb5	19.2	40.2	1.08	1.42E-216	7.41E-218
G0s2	13.3	27.7	1.08	1.22E-47	2.79E-48
Thyn1	12.3	26.2	1.08	5.51E-49	1.22E-49
Anxa1	11.7	24.3	1.08	3.11E-69	5.12E-70
Npm3	9	19	1.08	9.10E-34	2.81E-34
Msrb2	4.8	10.3	1.08	8.38E-26	3.22E-26
Rftn1	4.3	8.9	1.08	1.13E-52	2.34E-53
Myo1e	3.3	7	1.08	4.51E-69	7.45E-70
Tcaf1	3.3	6.7	1.08	3.30E-72	5.23E-73
Prcp	2.3	4.9	1.08	4.28E-31	1.43E-31
Trim68	1.8	3.9	1.08	5.28E-20	2.47E-20
Ddx58	1.3	2.8	1.08	3.86E-29	1.35E-29
Six5	1.1	2.2	1.08	1.54E-14	9.13E-15
Stxbp1	0.9	1.9	1.08	3.34E-16	1.82E-16
D8Ertd82e	0.7	1.5	1.08	1.28E-15	7.19E-16
Zfp57	0.5	1.3	1.08	6.40E-07	6.05E-07
Syce2	0.6	1.2	1.08	0.000859645	0.001117822
Prss23	29.3	61.2	1.07	9.22E-241	4.43E-242
Cd93	6.9	14.4	1.07	2.58E-191	1.55E-192
Csrp2	5.8	11.9	1.07	2.57E-22	1.11E-22
Spa17	3.5	7.3	1.07	4.08E-11	2.90E-11
Cnp	3.2	6.5	1.07	2.16E-30	7.35E-31
Hilpda	1.8	3.7	1.07	1.60E-08	1.34E-08
Ccdc189	1.7	3.4	1.07	2.71E-16	1.47E-16
Pstpip1	1.4	2.8	1.07	1.11E-09	8.62E-10
4930402H24Rik	1.2	2.5	1.07	3.35E-32	1.08E-32
Ddr2	1.2	2.5	1.07	5.35E-43	1.33E-43
Pdlim2	1.1	2.2	1.07	5.05E-08	4.39E-08
Mpped2	0.9	1.9	1.07	3.45E-11	2.44E-11

Ptprc	0.9	1.9	1.07	1.31E-19	6.21E-20
Cys1	0.8	1.6	1.07	3.06E-09	2.44E-09
Cenpp	0.7	1.5	1.07	0.000360804	0.000447619
Plek	0.7	1.5	1.07	3.18E-14	1.91E-14
Trim14	0.6	1.2	1.07	2.92E-09	2.33E-09
Tmem260	0.5	1.1	1.07	4.05E-13	2.57E-13
Oxct1	47.7	99.6	1.06	0	0
Hfe	7.2	15.9	1.06	2.60E-72	4.11E-73
Lman2l	6.9	14.6	1.06	1.85E-71	2.96E-72
Cdk10	5.5	11.1	1.06	7.48E-35	2.24E-35
Ly86	4.6	9.4	1.06	1.94E-19	9.27E-20
Creld2	3.2	6.6	1.06	4.70E-19	2.27E-19
Sh2d6	2.9	6.2	1.06	5.52E-19	2.67E-19
Nab2	3	6.1	1.06	5.13E-32	1.66E-32
BC017158	1.3	2.6	1.06	1.86E-13	1.16E-13
Gstt3	1	2	1.06	9.28E-09	7.66E-09
Ap3m2	0.9	1.9	1.06	2.14E-14	1.28E-14
Ripply3	0.9	1.8	1.06	6.48E-07	6.13E-07
Map3k5	0.8	1.6	1.06	2.44E-18	1.21E-18
Susd2	0.8	1.6	1.06	8.83E-11	6.39E-11
Ncaph	0.5	1.1	1.06	3.14E-07	2.89E-07
H2-K1	47.1	96.7	1.05	0	0
Anp32a	11.9	24.5	1.05	3.38E-99	3.92E-100
Cfd	6.6	13.7	1.05	2.99E-25	1.17E-25
Jkamp	6.2	12.9	1.05	4.17E-35	1.24E-35
Gypc	5.6	12	1.05	6.52E-53	1.35E-53
C130074G19Rik	5.8	11.9	1.05	8.35E-67	1.42E-67
2700094K13Rik	5.4	11.1	1.05	8.68E-16	4.84E-16
Tsen15	4.3	8.9	1.05	9.29E-21	4.23E-21
Id2	4.1	8.4	1.05	5.98E-22	2.62E-22
Irgm2	3.2	6.5	1.05	4.98E-46	1.16E-46
1190002N15Rik	2.1	4.4	1.05	3.84E-35	1.15E-35
Itga6	2.1	4.3	1.05	2.57E-51	5.45E-52
Tusc3	1.6	3.3	1.05	2.20E-11	1.54E-11
Vcam1	1.6	3.2	1.05	1.24E-22	5.33E-23
Gm10408	1.1	2.2	1.05	4.57E-08	3.96E-08
Wdr25	1	2.1	1.05	4.04E-12	2.70E-12
Blnk	1	2.1	1.05	4.42E-10	3.34E-10
Hist1h1e	0.8	1.7	1.05	0.000571148	0.000727546
Ifih1	0.7	1.5	1.05	1.60E-12	1.05E-12
Tdrp	0.7	1.5	1.05	1.25E-08	1.04E-08
Myl12b	19.3	39.4	1.04	5.62E-70	9.20E-71
Hsp90aa1	18.6	37.8	1.04	1.29E-206	7.07E-208
Irf9	3.1	6.4	1.04	1.88E-30	6.39E-31
Ggct	2.8	5.3	1.04	3.29E-12	2.19E-12
Plat	1.1	2.3	1.04	8.49E-13	5.48E-13
Apitd1	0.7	1.5	1.04	0.000413896	0.0005177
Ccr2	0.7	1.4	1.04	7.73E-11	5.58E-11
Itgam	0.5	1.1	1.04	4.21E-11	2.99E-11

St8sia4	0.6	1.1	1.04	8.86E-13	5.73E-13
Tagln2	21.6	43.6	1.03	3.44E-113	3.49E-114
Atpif1	21.1	42.9	1.03	3.38E-122	3.16E-123
Ache	15	30.7	1.03	9.44E-128	8.54E-129
Smim8	14.1	29.7	1.03	9.78E-44	2.41E-44
Pdia6	12.2	24.7	1.03	6.93E-99	8.07E-100
Pld2	4.5	9.3	1.03	3.16E-61	5.77E-62
Zfand1	4.2	8.5	1.03	1.57E-27	5.74E-28
Sri	3.2	6.6	1.03	1.10E-31	3.61E-32
Nrarp	2.9	5.9	1.03	1.07E-29	3.70E-30
Tmem106c	2.6	5.3	1.03	5.23E-16	2.89E-16
Fbln7	2.5	5	1.03	1.35E-28	4.82E-29
Metrnl	2	4	1.03	3.92E-20	1.82E-20
Tnfsf13	1.1	2.2	1.03	2.89E-08	2.48E-08
Tnfaip6	0.7	1.4	1.03	1.36E-05	1.44E-05
Gm13251	0.7	1.4	1.03	2.72E-11	1.91E-11
Fndc4	0.7	1.3	1.03	4.62E-07	4.32E-07
Snurf	0.6	1.3	1.03	5.30E-06	5.42E-06
Tbxas1	0.5	1.1	1.03	2.23E-05	2.42E-05
Crip1	45.7	91.8	1.02	2.00E-74	3.07E-75
Cntfr	7.8	16.5	1.02	1.59E-62	2.84E-63
Tmem141	7.6	16	1.02	2.87E-26	1.09E-26
Nkain1	7.4	15	1.02	5.24E-71	8.43E-72
Pdlim4	5.5	11	1.02	3.46E-25	1.36E-25
Pfdn4	5.5	11	1.02	1.55E-17	7.99E-18
Unc119	4.4	8.9	1.02	4.16E-20	1.94E-20
Cav2	3.7	7.5	1.02	1.35E-38	3.72E-39
Kbtbd13	3.5	7.1	1.02	7.42E-40	1.99E-40
Anapc10	4	6.8	1.02	4.03E-16	2.21E-16
Jam3	3.4	6.7	1.02	7.21E-26	2.76E-26
Ankmy2	1.8	3.7	1.02	2.53E-18	1.26E-18
Heyl	1.7	3.5	1.02	1.56E-30	5.27E-31
Rsad2	1.6	3.1	1.02	3.17E-23	1.33E-23
Sdc1	1.3	2.6	1.02	1.01E-15	5.65E-16
Umps	1	2.1	1.02	6.15E-14	3.74E-14
Drp2	0.9	1.8	1.02	2.62E-23	1.09E-23
Naalad2	0.6	1.3	1.02	2.02E-09	1.59E-09
Pvalb	6499.4	13157.7	1.01	0	0
Col4a1	29.6	59.1	1.01	0	0
Tmx2	16.5	33.9	1.01	2.42E-128	2.19E-129
Emc9	14.5	29.2	1.01	6.00E-42	1.54E-42
Dcakd	12.8	26.9	1.01	1.47E-87	1.94E-88
Atp1b4	10.9	21.7	1.01	1.36E-158	9.95E-160
Bcs1l	5.4	10.9	1.01	1.16E-37	3.27E-38
Nrp1	4.5	8.9	1.01	4.49E-96	5.39E-97
Sdc3	4	8.1	1.01	8.50E-72	1.35E-72
C1qtnf5	2.9	6.1	1.01	9.24E-16	5.16E-16
Antxr1	2.7	5.3	1.01	2.60E-52	5.46E-53
Prrx2	1.6	3.2	1.01	3.27E-08	2.82E-08

Amhr2	1.5	2.9	1.01	1.59E-11	1.10E-11
Arhgef9	1	2.2	1.01	3.06E-22	1.33E-22
Ccnb2	1	2	1.01	5.90E-07	5.57E-07
Ly6c2	0.9	1.9	1.01	0.00021237	0.00025608

Table S2 Downregulated genes in Klf15KO muscles at Day 7 after CTX injection

Gene	WT CTX D7 Average FPKM	Klf15KO CTX D7 Average FPKM	log2 (Klf15KO/WTD7)	Qvalue (WT-vs-Klf15KO)	Pvalue (WT-vs-Klf15KO)
Serpina3m	1.1	0	-6.38	6.72E-21	3.04E-21
Serpina3n	50.9	1.1	-5.5	0	0
Doc2b	1.8	0.1	-4.88	2.19E-102	2.45E-103
Fkbp5	60.4	2.1	-4.82	0	0
Adhfe1	4.6	0.2	-4.68	2.53E-111	2.62E-112
Hif3a	1.1	0	-4.55	5.64E-80	8.03E-81
Bmp8a	1.2	0.1	-4.53	2.35E-32	7.53E-33
Lvrn	1.4	0.1	-4.42	4.06E-67	6.85E-68
Arrdc2	62.9	3.8	-4.14	0	0
Lcn2	3.4	0.2	-4.1	2.03E-30	6.90E-31
Gck	21.3	1.3	-4.07	0	0
Cyp2e1	2.4	0.2	-3.98	1.39E-43	3.44E-44
Fam107a	2.6	0.2	-3.97	3.42E-83	4.72E-84
Prodh	8.5	0.6	-3.81	1.60E-187	9.74E-189
Angptl7	19.9	1.5	-3.73	0	0
Acot1	1.9	0.2	-3.52	5.64E-29	1.99E-29
Cebpd	75.9	6.9	-3.45	0	0
Slc10a6	3.8	0.4	-3.45	1.01E-64	1.77E-65
Card14	2.4	0.2	-3.34	2.62E-81	3.66E-82
Bpifb1	1	0.1	-3.29	1.50E-15	8.47E-16
Il6ra	4.3	0.5	-3.22	6.33E-124	5.85E-125
Mt2	626	70.8	-3.14	0	0
Slco4a1	1.7	0.2	-3.14	1.95E-33	6.08E-34
Rnase13	3.1	0.4	-3.08	4.76E-33	1.50E-33
Gabrr2	7.2	0.9	-3.04	4.99E-107	5.42E-108
BC048679	1.8	0.2	-3	8.41E-11	6.08E-11
Cth	1.2	0.2	-2.94	9.12E-18	4.66E-18
Fmo2	11.2	1.5	-2.93	0	0
Map3k6	5	0.6	-2.87	3.16E-170	2.17E-171
Scgb3a1	1	0.2	-2.75	1.27E-05	1.35E-05
Inmt	3.8	0.6	-2.74	6.74E-29	2.39E-29
Prom1	2.2	0.3	-2.74	3.55E-55	7.10E-56
Chac1	39.2	6	-2.72	0	0
Mt1	494.4	76.4	-2.69	0	0
Fbxo32	93.1	14.7	-2.68	0	0
Cep85l	5.1	0.7	-2.67	5.77E-152	4.37E-153
Trim63	327.1	51.7	-2.66	0	0
Ddit4	42.1	6.6	-2.66	0	0
Cldn15	6.4	1	-2.66	1.24E-85	1.68E-86
8430408G22Rik	28.9	4.6	-2.65	2.09E-264	9.35E-266
S100a8	4	0.6	-2.65	1.38E-11	9.49E-12
Spock2	5.6	0.9	-2.62	1.34E-191	7.98E-193

Aldh1a1	54.1	9.2	-2.58	0	0
Rtn4rl2	1	0.2	-2.55	1.09E-14	6.38E-15
Per1	36.4	6.4	-2.5	0	0
Plin5	12.3	2.2	-2.5	1.65E-155	1.23E-156
Myoc	11	2	-2.5	1.22E-152	9.16E-154
Tac4	1.9	0.3	-2.45	4.70E-16	2.58E-16
Gm29801	1.2	0.2	-2.43	3.60E-19	1.73E-19
Tppp2	1.9	0.4	-2.41	6.11E-10	4.66E-10
Maff	5.7	1.1	-2.39	7.12E-66	1.22E-66
Gnmt	1.4	0.3	-2.39	2.41E-10	1.79E-10
Rasd1	2.7	0.5	-2.38	5.64E-28	2.04E-28
E130012A19Rik	1.4	0.3	-2.38	1.22E-21	5.38E-22
Stc1	1.7	0.3	-2.36	3.15E-40	8.36E-41
Plin4	67.9	13.8	-2.32	0	0
Fbp2	24.8	5.3	-2.25	2.51E-185	1.54E-186
Acss1	8.5	1.8	-2.23	7.81E-178	5.04E-179
Syt12	7.1	1.5	-2.21	1.22E-133	1.06E-134
Capn11	1.6	0.4	-2.2	1.79E-21	7.97E-22
Glul	366.5	80.1	-2.19	0	0
Fam134b	147.3	32.6	-2.19	0	0
Cish	4.1	1	-2.16	5.42E-71	8.74E-72
Ibsp	3.4	0.8	-2.15	2.02E-37	5.75E-38
Inhbb	1.2	0.3	-2.15	2.48E-29	8.64E-30
Tmem252	11.2	2.5	-2.14	1.62E-151	1.23E-152
Pik3r1	16.5	4	-2.13	0	0
Kcnk5	1.3	0.3	-2.13	8.43E-26	3.24E-26
Tfdp2	21.8	5.2	-2.12	0	0
Sorbs1	68	16.3	-2.11	0	0
Cdhr3	2.6	0.7	-2.11	6.52E-40	1.74E-40
Gm7160	2.7	0.6	-2.1	3.53E-08	3.04E-08
1700066C05Rik	3.9	0.9	-2.09	4.46E-09	3.60E-09
Dpep1	8.3	2	-2.08	8.66E-97	1.03E-97
Fzd4	7.3	1.8	-2.08	9.02E-144	7.32E-145
Ppp1r15a	43.1	10.2	-2.07	0	0
Scara5	4.2	1	-2.07	2.26E-85	3.05E-86
Pdk4	152.7	37.5	-2.05	0	0
Clec4d	2.4	0.6	-2.05	1.63E-17	8.40E-18
Wisp2	2.1	0.5	-2.01	5.06E-20	2.36E-20
Irs2	3.3	0.8	-2	7.35E-69	1.22E-69
Mmp13	15.2	3.8	-1.99	1.16E-202	6.50E-204
Klk1b26	5.8	1.5	-1.98	4.12E-27	1.52E-27
Car4	13.2	3.4	-1.97	3.18E-85	4.31E-86
Tango2	24.5	5.6	-1.96	2.38E-202	1.35E-203
Npc1	19.6	5.2	-1.93	0	0
Ednrb	8.2	2.3	-1.93	5.44E-149	4.19E-150
Cyp26b1	2	0.5	-1.93	2.32E-46	5.39E-47
Iqck	1.1	0.3	-1.93	4.01E-12	2.68E-12
Slc43a1	10.3	2.5	-1.91	6.62E-130	5.92E-131
Fxyd2	7.3	2	-1.88	8.97E-20	4.23E-20

Usp54	5.9	1.5	-1.85	7.69E-160	5.59E-161
Apod	58.6	16.4	-1.84	0	0
Jade2	1.7	0.5	-1.83	6.92E-47	1.59E-47
Zdhhc23	1.5	0.4	-1.83	3.04E-39	8.27E-40
Gm826	1.5	0.4	-1.83	1.93E-07	1.75E-07
Cd163	2	0.6	-1.82	9.31E-39	2.57E-39
Igfbp3	30.9	8.8	-1.8	0	0
Vegfd	7.1	2	-1.8	1.69E-57	3.26E-58
Pnpla2	372.5	107.6	-1.79	0	0
Ppp1r1a	12.5	3.7	-1.79	2.79E-70	4.53E-71
Pnpla3	8.8	2.6	-1.79	8.77E-173	5.92E-174
Ehd3	5.8	1.7	-1.78	1.18E-92	1.48E-93
Tmem82	2.3	0.7	-1.78	6.75E-22	2.96E-22
Net1	39.8	11.6	-1.77	0	0
Slc25a34	6.4	1.9	-1.77	5.25E-75	8.02E-76
Fam209	1.1	0.3	-1.77	0.000170043	0.000203181
Galnt15	7.3	2.2	-1.75	2.17E-206	1.20E-207
Foxred2	1.4	0.4	-1.74	1.62E-33	5.05E-34
Nfil3	36.6	10.8	-1.73	4.28E-296	1.78E-297
Apoc2	4	1.2	-1.73	6.89E-11	4.96E-11
Eif4ebp1	158.7	48.1	-1.72	0	0
Rasd2	3.4	1	-1.72	3.55E-40	9.42E-41
Atoh8	10.6	3.2	-1.71	8.40E-102	9.46E-103
Gda	4.6	1.4	-1.7	2.39E-96	2.86E-97
Xdh	12.4	4.1	-1.69	7.64E-173	5.14E-174
Lnx1	1.2	0.4	-1.69	2.76E-14	1.66E-14
Ctxn3	39.4	12.3	-1.68	6.80E-213	3.64E-214
Cpm	1	0.3	-1.68	1.65E-21	7.33E-22
Kcnj12	22.2	7.1	-1.66	0	0
Slc39a14	7.9	2.6	-1.66	3.02E-142	2.48E-143
Gramd3	4.9	1.6	-1.66	6.42E-51	1.37E-51
Arntl	11.1	3.6	-1.65	7.22E-125	6.64E-126
Acer2	4.1	2.2	-1.65	1.87E-39	5.06E-40
Hsd17b14	1.4	0.5	-1.65	2.89E-07	2.66E-07
Slc38a2	88.4	28.2	-1.64	0	0
Csrnp1	3.7	1.2	-1.64	2.65E-43	6.58E-44
Fas	3.5	1.1	-1.64	5.58E-21	2.52E-21
Sema3g	3.2	1	-1.64	3.22E-55	6.44E-56
Pim3	24.9	8	-1.63	4.47E-229	2.26E-230
Selenbp1	4.7	1.5	-1.63	5.37E-31	1.80E-31
Nr2c2	1.9	0.6	-1.63	1.36E-55	2.68E-56
Edn1	1	0.3	-1.63	9.31E-10	7.19E-10
Fbxo31	69.6	22.7	-1.62	0	0
Mcf2l	6.8	2.2	-1.62	4.03E-135	3.46E-136
Trp53inp1	9.4	3.1	-1.61	1.69E-189	1.02E-190
Gm40814	2.9	0.9	-1.61	5.75E-29	2.03E-29
Llgl2	1.7	0.6	-1.6	1.76E-23	7.32E-24
Aldh6a1	28.5	9.5	-1.59	0	0
Filip1	27.6	9.5	-1.58	0	0

Cnksr1	122.9	42.7	-1.57	0	0
Foxo1	11.7	3.9	-1.57	8.74E-234	4.33E-235
Tbx6	3.5	1.2	-1.57	3.68E-23	1.55E-23
Prima1	1.5	0.6	-1.57	4.50E-21	2.02E-21
Shd	1.1	0.4	-1.57	3.78E-08	3.26E-08
Dusp26	28.5	10	-1.56	1.14E-178	7.32E-180
Crybb3	4.6	2	-1.56	6.07E-24	2.49E-24
Tmc7	3.5	1.2	-1.56	2.95E-58	5.61E-59
Lrrc52	2.1	0.7	-1.56	3.10E-10	2.31E-10
Tacc2	277.2	98.7	-1.55	0	0
Rora	12.4	4.7	-1.55	0	0
Slc43a3	19	6.4	-1.54	4.64E-160	3.35E-161
Hrct1	4.7	1.6	-1.54	2.55E-16	1.39E-16
Rbm15	4.1	1.4	-1.53	2.54E-47	5.82E-48
Tob2	44.1	15.3	-1.52	0	0
Wdr62	1.7	0.6	-1.52	7.70E-30	2.65E-30
Retnla	7.4	2.6	-1.51	1.07E-15	6.02E-16
Diras1	1.4	0.5	-1.51	3.03E-15	1.74E-15
Mss51	68.6	24.6	-1.49	0	0
Spsb1	10.5	3.7	-1.49	3.27E-108	3.52E-109
Arl5c	4.6	1.5	-1.49	5.76E-23	2.44E-23
Hook1	2.6	0.9	-1.49	5.25E-37	1.51E-37
Acot2	13.1	4.7	-1.48	4.72E-96	5.68E-97
Ankrd9	146.1	52.6	-1.47	0	0
Flt1	7.3	2.7	-1.46	5.57E-149	4.30E-150
Duxbl1	1.1	0.4	-1.46	3.42E-09	2.73E-09
Art3	167.4	62.1	-1.45	0	0
Ppp1r3c	163.7	59.3	-1.45	0	0
Kyat3	5.6	2.1	-1.45	3.44E-39	9.37E-40
Tns1	68.4	25.5	-1.44	0	0
Mocs1	16.4	6.4	-1.44	9.31E-124	8.61E-125
Rilpl1	118.6	44.1	-1.43	0	0
Rbl2	11.5	4.3	-1.43	3.41E-176	2.22E-177
Anxa8	1.2	0.5	-1.42	1.30E-08	1.08E-08
Tsc22d3	111.4	40.9	-1.41	0	0
Sesn1	23.7	9	-1.41	2.98E-195	1.77E-196
Zbtb16	28.2	10.6	-1.4	0	0
Usp53	4.5	1.7	-1.4	5.87E-84	8.05E-85
Klf9	68.5	26.2	-1.39	0	0
Mafk	16.2	6.2	-1.38	1.39E-137	1.18E-138
Nr4a3	5.2	2	-1.38	2.83E-87	3.75E-88
Rps6ka5	3.5	1.3	-1.38	8.94E-43	2.25E-43
Socs3	4.9	1.9	-1.37	3.22E-41	8.41E-42
Rnf125	4.7	1.8	-1.37	9.66E-76	1.46E-76
Agfg2	3.1	1.2	-1.37	1.71E-28	6.14E-29
Ern1	1.8	0.7	-1.37	2.04E-22	8.80E-23
Ube4a	10.5	4.1	-1.36	1.67E-185	1.02E-186
Cp	1.8	0.7	-1.36	9.06E-26	3.49E-26
Elk4	1.3	0.5	-1.36	8.98E-18	4.58E-18

Ttll7	13.6	5.4	-1.35	3.95E-287	1.69E-288
Fasn	4.1	1.6	-1.35	2.13E-118	2.06E-119
Zbtb8b	1.7	0.7	-1.35	3.66E-23	1.54E-23
Dusp4	1.4	0.6	-1.35	3.41E-11	2.41E-11
Cited2	77	30.5	-1.34	0	0
Wbp11	37.8	14.9	-1.34	0	0
Tns2	18.3	7.2	-1.34	5.76E-241	2.76E-242
Slc15a4	10.8	4.4	-1.34	4.29E-81	6.05E-82
Map3k8	2	0.8	-1.34	2.48E-13	1.56E-13
1700067K01Rik	1.1	0.4	-1.34	5.48E-06	5.61E-06
1700109H08Rik	7.2	2.9	-1.33	8.00E-16	4.45E-16
Slit3	1.2	0.5	-1.33	2.45E-18	1.22E-18
Hectd1	35.4	14.1	-1.32	0	0
Lrg1	11.8	4.7	-1.32	8.28E-46	1.94E-46
Zbed5	64.5	24.4	-1.31	3.59E-81	5.03E-82
Art5	31.5	13	-1.31	4.64E-147	3.64E-148
Rhbdf2	16.1	6.7	-1.31	4.92E-140	4.11E-141
Cblb	6	2.4	-1.31	2.42E-108	2.60E-109
Gm11100	4.5	1.8	-1.31	2.82E-08	2.41E-08
Penk	3.9	1.6	-1.31	1.41E-16	7.57E-17
Igfn1	34.5	15.8	-1.3	0	0
Nrbp2	31.3	12.8	-1.3	1.24E-227	6.27E-229
Tmem100	9.1	3.6	-1.3	1.41E-37	3.99E-38
Synj2	20.2	8.2	-1.29	1.54E-302	6.28E-304
Klhl38	13.4	5.6	-1.29	2.32E-80	3.30E-81
Zfyve1	11.2	4.9	-1.28	5.57E-106	6.08E-107
Ddi2	5.7	2.4	-1.28	1.38E-54	2.77E-55
Gfpt2	2.8	1.2	-1.28	9.51E-24	3.93E-24
Abcb1b	1.5	0.6	-1.28	9.70E-19	4.74E-19
Snai3	16.9	7	-1.27	6.17E-71	9.97E-72
Lrrc58	11.3	4.6	-1.27	1.28E-255	5.89E-257
Fam117b	2.9	1.2	-1.27	5.48E-43	1.37E-43
Gucd1	32.1	13.5	-1.26	1.09E-237	5.35E-239
Rorc	28.3	12.9	-1.26	6.64E-167	4.60E-168
Ctgf	12.4	5.1	-1.26	4.98E-78	7.25E-79
Glt28d2	5.8	2.4	-1.25	1.54E-41	3.98E-42
Pygo1	3	1.3	-1.25	8.60E-15	5.03E-15
1110017D15Rik	1	0.7	-1.25	0.000386917	0.000481662
Ccdc6	29.5	12.7	-1.24	0	0
Sh3rf2	7.8	3.4	-1.24	3.95E-94	4.85E-95
Sbsn	3.4	1.5	-1.24	3.85E-15	2.21E-15
Spidr	1.2	0.5	-1.24	7.80E-11	5.63E-11
Cebpb	99.4	42.4	-1.23	0	0
Ank3	35.8	15.7	-1.23	0	0
Ldlrad3	10.2	4.3	-1.23	8.64E-98	1.01E-98
Npr3	3.3	1.4	-1.23	7.14E-55	1.43E-55
Sorbs3	48	23.2	-1.22	2.66E-283	1.17E-284
Fbxl20	6.3	2.7	-1.22	3.84E-131	3.38E-132
Ucn2	3	1.3	-1.22	1.33E-08	1.11E-08

Gsn	113.7	49.5	-1.21	0	0
Asb11	85.8	37.8	-1.21	0	0
Mn1	16.7	7.3	-1.21	5.83E-249	2.69E-250
Ccdc57	7.8	3.4	-1.21	2.92E-57	5.64E-58
Adam8	4.3	1.8	-1.21	3.69E-34	1.13E-34
Ms4a4d	2.7	1.2	-1.21	3.84E-11	2.72E-11
Asic3	2.2	0.9	-1.21	1.20E-25	4.65E-26
Dnm3	1.4	0.7	-1.21	8.04E-17	4.26E-17
Cd36	65.1	27.9	-1.2	0	0
Slc6a6	32.9	14.3	-1.2	0	0
Acvr1b	11.8	5.2	-1.2	1.45E-93	1.80E-94
Ubc	752.6	330.1	-1.19	0	0
Rhou	6.1	2.7	-1.19	2.59E-49	5.70E-50
Kcnc3	3	1.3	-1.19	5.11E-42	1.31E-42
Spns2	11.9	5.5	-1.18	2.37E-88	3.09E-89
Abca7	6	2.7	-1.18	3.87E-89	5.01E-90
Slc6a9	4.1	1.8	-1.18	8.36E-32	2.72E-32
Itgbl1	3.8	1.7	-1.18	4.57E-22	1.99E-22
Mmp3	2.5	1.1	-1.18	7.28E-12	4.94E-12
Cntf	2.1	0.9	-1.18	3.20E-07	2.95E-07
4933415F23Rik	1.5	0.7	-1.18	1.21E-07	1.08E-07
Fam124a	1.4	0.6	-1.18	1.03E-12	6.71E-13
Serinc3	130.8	57.7	-1.17	0	0
Gpx3	310.7	137.8	-1.16	0	0
Ipo13	110.5	49.6	-1.16	0	0
Dgat2	15.3	6.9	-1.16	4.01E-79	5.75E-80
Osgin1	10	4.4	-1.16	8.06E-46	1.89E-46
Foxk1	5.6	2.6	-1.16	8.83E-94	1.09E-94
Crebrf	3.5	1.6	-1.16	8.79E-61	1.62E-61
Armcx5	3.5	1.5	-1.16	1.94E-23	8.09E-24
Nfxl1	1.9	0.9	-1.16	2.53E-17	1.31E-17
Slpi	1.3	0.6	-1.16	0.000391799	0.000488275
Ifitm1	2.8	1.3	-1.15	2.31E-05	2.51E-05
Aoc2	1.5	0.7	-1.15	8.34E-11	6.03E-11
Lmod2	370.2	166.7	-1.14	0	0
Cry2	15.7	7.1	-1.14	7.64E-141	6.33E-142
Col7a1	8.1	3.6	-1.14	2.96E-166	2.06E-167
Msc	4	1.9	-1.14	2.44E-17	1.26E-17
Acadl	304.9	138.7	-1.13	0	0
Rere	25.9	11.7	-1.13	0	0
Trpv4	1.2	0.5	-1.13	9.83E-10	7.60E-10
Ybx3	1247.1	578.9	-1.12	0	0
Pnrc1	112	51.4	-1.12	0	0
Dusp1	69.6	31.9	-1.12	2.29E-289	9.70E-291
Ulk1	26.6	12.7	-1.12	3.53E-279	1.56E-280
Pde4d	25.9	11.4	-1.12	0	0
Pim1	23.7	10.8	-1.12	3.03E-89	3.92E-90
Olfr1033	13	5.9	-1.12	1.40E-74	2.15E-75
Smyd2	250.4	117.9	-1.11	0	0

Anxa11	131.6	60.9	-1.11	0	0
Oat	82.2	38.2	-1.11	0	0
Tpbgl	54.2	25.3	-1.11	0	0
Mlycd	48.1	22.9	-1.11	1.02E-213	5.45E-215
Sec24a	6.6	3.2	-1.11	1.76E-90	2.25E-91
Zfp966	2.1	1	-1.11	1.46E-10	1.07E-10
Zfp967	2.1	1	-1.11	1.67E-10	1.23E-10
Dfna5	1.3	0.6	-1.11	1.31E-07	1.18E-07
Pde4dip	364.1	171.4	-1.1	0	0
Tmem52	221.9	104	-1.1	0	0
Gm39972	18.4	8.2	-1.1	2.66E-73	4.14E-74
Hipk1	8.1	3.8	-1.1	1.64E-131	1.44E-132
Ifi205	6.4	2.9	-1.1	1.21E-22	5.19E-23
Zrsr1	4.5	2.1	-1.1	3.02E-43	7.50E-44
Chil1	1.8	0.8	-1.1	1.11E-07	9.89E-08
Arhgap36	1.3	0.6	-1.1	3.44E-09	2.75E-09
Egln3	47	21.9	-1.09	1.18E-257	5.38E-259
Idh1	20.5	9.6	-1.09	7.25E-96	8.75E-97
D230025D16Rik	13.5	6.4	-1.09	3.86E-81	5.42E-82
Cdc14a	7.8	3.5	-1.09	2.26E-68	3.78E-69
Jmy	5.1	2.4	-1.09	7.00E-94	8.63E-95
Hoga1	3.4	1.6	-1.09	1.04E-12	6.73E-13
Ppara	3.3	1.5	-1.09	9.32E-51	2.00E-51
Pdlim5	434.7	207.3	-1.08	0	0
Ctsl	133	62.5	-1.08	0	0
St3gal5	53.9	25.9	-1.08	1.50E-242	7.13E-244
Nfkbia	45.1	21.2	-1.08	4.97E-144	4.01E-145
Klf15	34	15.8	-1.08	4.48E-171	3.06E-172
Zkscan17	11.6	5.5	-1.08	8.66E-88	1.14E-88
Dusp7	9.4	4.4	-1.08	1.13E-62	2.02E-63
Arl4a	7	3.3	-1.08	4.11E-53	8.47E-54
Smcr8	5	2.4	-1.08	2.62E-76	3.90E-77
Gpr19	2.6	1	-1.08	1.65E-11	1.14E-11
Bnip3	144	68.6	-1.07	0	0
Lrrc30	98.4	46.4	-1.07	0	0
Nos1	16.2	7.8	-1.07	0	0
Spen	4.6	2.2	-1.07	1.40E-114	1.40E-115
Lpin1	52.8	25.6	-1.06	0	0
Klf13	14.6	7	-1.06	2.86E-185	1.76E-186
Clk2-scamp3	5.6	2.7	-1.06	2.69E-33	8.41E-34
Mbp	2.7	1.2	-1.06	1.82E-14	1.08E-14
Car15	2.1	1	-1.06	1.68E-06	1.65E-06
Abcb1a	1.8	0.9	-1.06	8.58E-19	4.18E-19
Mylk2	352.5	183.3	-1.05	0	0
Tmem38b	46.9	22.6	-1.05	3.48E-258	1.58E-259
Ocrl	6.1	3	-1.05	9.05E-64	1.60E-64
Nova1	2	1.1	-1.05	2.16E-14	1.29E-14
Zfp189	1.6	0.8	-1.05	1.72E-10	1.27E-10
Tmem198	1.1	0.5	-1.05	5.31E-06	5.43E-06

Psme4	41.8	20.4	-1.04	0	0
Osmr	4.3	2	-1.04	8.20E-39	2.26E-39
Zfp646	3.1	1.5	-1.04	1.44E-38	3.98E-39
Kcna5	2.9	1.4	-1.04	4.21E-18	2.12E-18
Avil	2.6	1.3	-1.04	6.17E-17	3.26E-17
Adgrf5	13.1	7.4	-1.03	1.21E-205	6.76E-207
Cbx7	8.9	4.4	-1.03	6.48E-51	1.39E-51
Bmp6	4	2	-1.03	8.28E-24	3.41E-24
Fdft1	21.6	10.7	-1.02	1.03E-148	7.94E-150
Gm10705	18.7	9.2	-1.02	3.69E-24	1.50E-24
4931406P16Rik	10.4	5.1	-1.02	5.73E-115	5.72E-116
Foxo3	8.4	4.1	-1.02	6.41E-107	6.98E-108
Slc2a1	6.9	3.4	-1.02	9.24E-34	2.86E-34
LOC102634078	5	2.5	-1.02	2.98E-20	1.38E-20
Vldlr	29.6	15	-1.01	0	0
Fam63a	28.6	16.7	-1.01	1.06E-124	9.76E-126
Alpk2	22.6	11.1	-1.01	1.93E-300	7.94E-302
Ece1	17.3	8.5	-1.01	8.10E-151	6.21E-152
Lgals4	6.4	3.2	-1.01	6.27E-21	2.83E-21

Table S3 Oligonucleotides

Gene	Application	Forward Sequence (5'- 3')	Reverse Sequence (5'- 3')
Klf15	qRT-PCR	GTCAACATCCAGGGGCAGACCTT	TTGGCGGCAATGGGCACAG
Myog	qRT-PCR	CAGCCCAGCGAGGGAATTTA	AGAAGCTCCTGAGTTTGCCC
Ckm	qRT-PCR	GGCAACACCCACAACAAGTTC	CCTTGAAGACCGTGTAGGACT
Pax7	qRT-PCR	GAATCAGAACCCGACCTCCC	CGCCGGTTACTGAACCAGA
Fkbp5	qRT-PCR	TATGCTTATGGCTCGGCTGG	GAGTATCCCTCGCCTTTCCG
Gck	qRT-PCR	GAGCCCAGTCGTTGACTCTG	CTTCTGAGCCTTCTGGGGTG
PPARa	qRT-PCR	CCTGAACATCGAGTGTCGAATAT	GTTCTTCTTCTGAATCTTGCAGC
Cebpd	qRT-PCR	AGAACCCGCGGCCTTCTAC	GTCGTACATGGCAGGAGTCG
Ddit4	qRT-PCR	CTGGTTACAGCCAGTCCCTG	GAGCCACCTGCATACAACCT
Bmp8a	qRT-PCR	TCTGGTCAGTACCACAGTAGC	AGAGGTGGCACTCAGTTTGG
Gapdh	qRT-PCR	AATGCATCCTGCACCACC	ATGCCAGTGAGCTTCCCG
Fkbp5-1	ChIP	GGGCCTTTCTCAGTGTGTGT	GAGAGGGGCTCTGACCTACC
Fkbp5-2	ChIP	CTGACTGTTCCCCACAGACAC	CTGATCGAACAGCTAGTGGCA
Fkbp5-3	ChIP	CCCACACATACCTCGTGCTT	GGGTCCTTCTCAGTCGCTTG
Fkbp5-4	ChIP	GACCTCTGTTGGGTACTGCACTC	CCTGGGGACTTGAACTCTAACT
Fkbp5-5	ChIP	AGTTAGAGTTCAAGTCCCCAGG	GGAACCCAAAGGATGGCTGTT
Fkbp5-6	ChIP	CAGTCAGCTTCCTCCATCCC	GTGCACTGCCTATGCAAATGA
KLF15-gRNA1	CRSPR-Cas9	caccgTCATCCTCCGAGATGGGCGA	aaacTCGCCCATCTCGGAGGATGAC
KLF15-gRNA2	CRSPR-Cas9	caccgTCTGCAGTAGCACCGGCACG	aaacCGTGCCGGTGCTACTGCAGAC

Table S4 Antibodies

Name	Application	Source
Anti-KLF15	Western blotting: 1:1,000<br>IF: 1:200	Abcam (ab185958)
Anti-Myogenin (F5D)	IF: 1:100	Abcam (ab1835)
Anti-Embryonic Myosin Heavy Chain	IF: 1:50	DSHB (developmental studies Hybridoma Bank), (F1.652), AB_528358
Anti-Myosin Heavy Chain	IF: 1:50<br>Western blotting: 1:1,000	DSHB (developmental studies Hybridoma Bank), (MF 20), AB_2147781
Anti-Laminin	IF: 1:200	Sigma (L9393)
Anti-Pax7	IF: 1: 50	DSHB (developmental studies Hybridoma Bank), AB_528428
Anti-Fkbp5	Western blotting: 1:1,000	Cell Signaling Technology (# 8245)
Anti-Flag	ChIP: 2ug/ChIP	Sigma
Histone	ChIP: 10ul/ChIP	Cell Signaling Technology (#4620)
Anti-WGA	IF: 1:100	Sigma (L4895)
Anti-GAPDH	Western blotting: 1:1,000	Cell Signaling Technology (#2118)
