# Supplementary material for: The transcriptional regulator KLF15 is necessary for myoblast differentiation and muscle regeneration by activating FKBP5
Source: J Biol Chem. 2023 Sep 4;299(10):105226. doi: 10.1016/j.jbc.2023.105226 (PMC10622842; doi:10.1016/j.jbc.2023.105226)
Supplement: Supporting Figures S1–S5 [file mmc2.pdf]

Supplementary figures

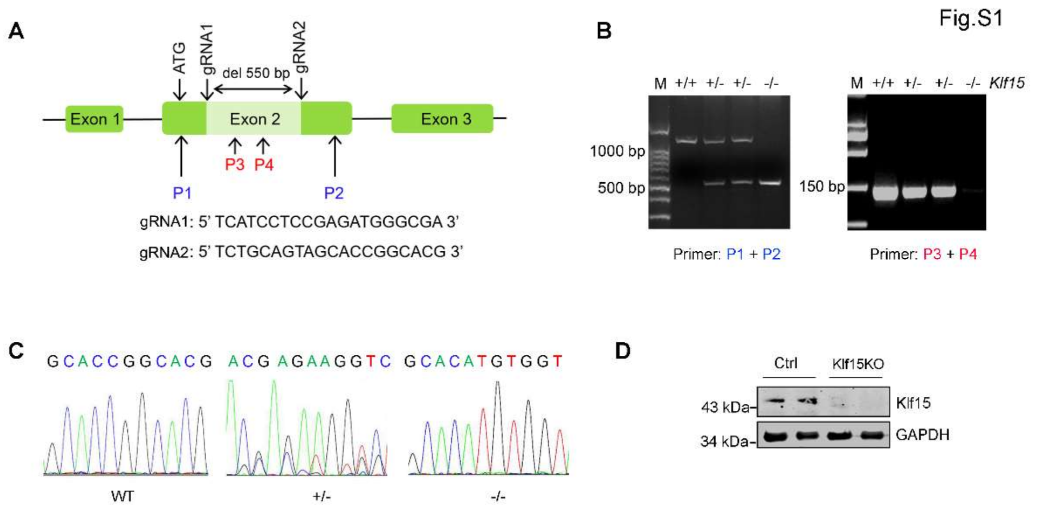

Figure S1. Generation of Klf15KO mice using CRISPR/Cas9 system. A. Schematic illustration of the location of the guide RNA (gRNA) target sequence in KLF15. **B.** PCR screening of Klf15KO newborns. **C.** Sequences of boundaries between the gRNA1 and gRNA2 target sites. **D.** Western blot analysis of KLF15 protein levels in the tibialis anterior (TA) muscle isolated from WT and Klf15KO mice.

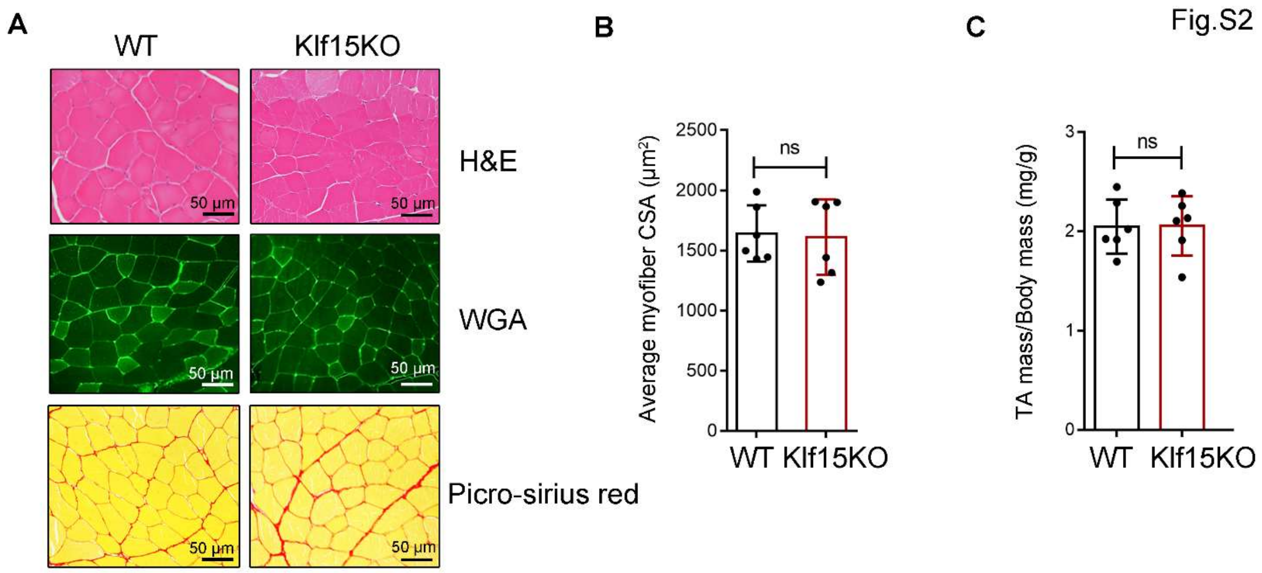

Figure S2. Skeletal phenotyping of Klf15KO mice. A. Representative H&E-stained sections (top panels), WGA-stained sections (middle panels), and Picro-Sirius red-stained sections (bottom panels) of TA muscles from WT and Klf15KO mice at baseline. **B.** Average cross-sectional area (CSA) of myofibers from WT and Klf15KO TA muscles. **C.** TA muscle weight was normalized to body weight (n=6, ns, no significant difference, unpaired student *t* test).

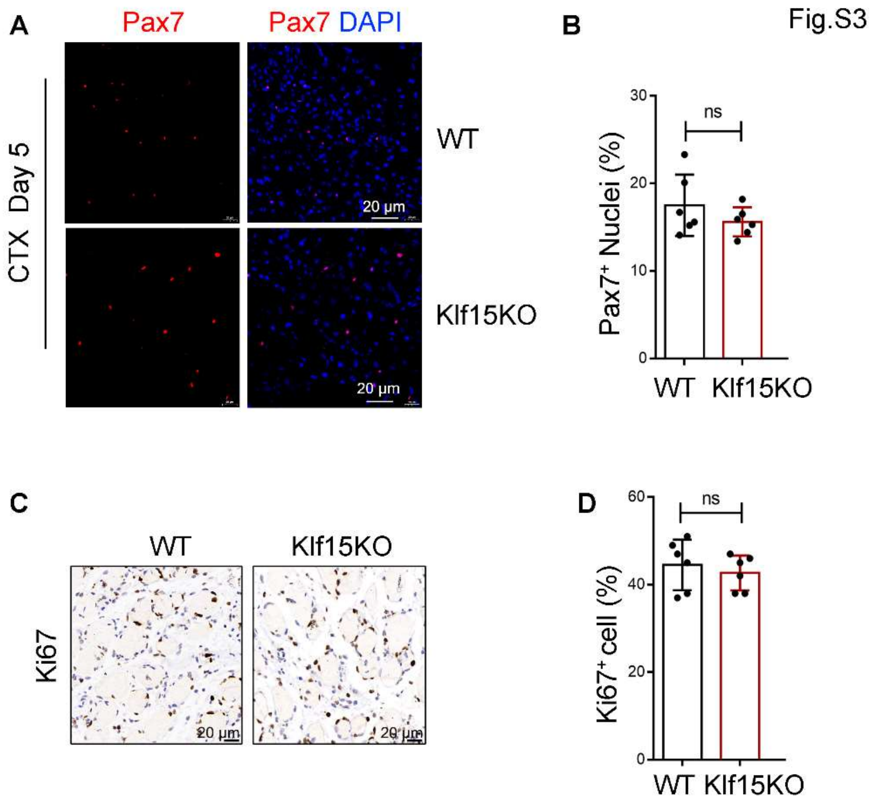

Figure S3. KLF15 had no effect on satellite cell proliferation. **A.** and **B.** Representative image of immunostaining for Pax7 (red) and DAPI (blue) in WT and Klf15KO TA muscles on day 5 after CTX injection and quantification of $Pax7^{+}$ cells per field (n=6, ns, no significant difference, unpaired student *t* test). **C.** and **D.** Representative images of Ki67 immunohistochemistry and quantitative measurement of $Ki67^{+}$ cells in WT and Klf15KO TA muscles on day 5 after CTX injection (n=6, ns, no significant difference, unpaired student *t* test).

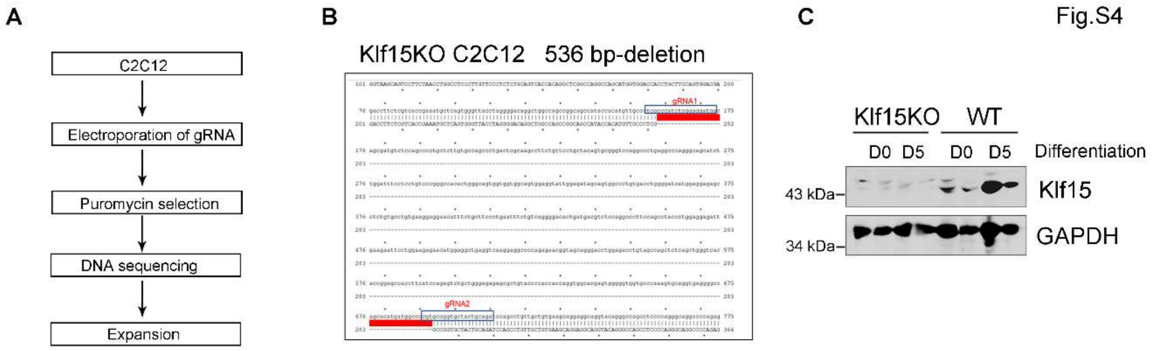

Figure S4. Establishment of Klf15KO myoblast cells using CRISPR-Cas9 system. **A.** C2C12 myoblasts were transfected with px459-Cas9-KLF15 gRNA plasmids by electroporation. After puromycin selection, the cells were cloned using limiting dilution. KLF15 deletion was confirmed by Sanger sequencing. **B.** Sanger sequencing indicates a 536-bp deletion mutation in KLF15. **C.** Western blot analysis of KLF15 protein levels in WT and Klf15KO C2C12 cells cultured in growth or differentiation medium for five days. GAPDH was used as a loading control.

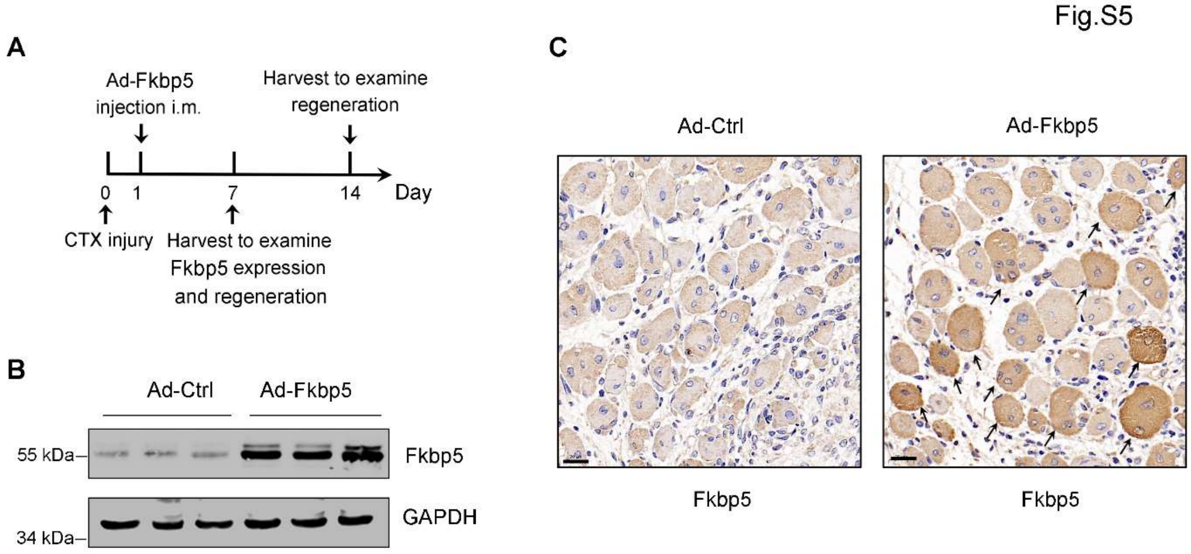

Figure S5. Overexpression of FKBP5 in Klf15KO muscle. A. Schematic representation of experimental design. **B.** The TA muscles of Klf15KO mice were injected with 30 μL (4×10^9 PFU) of adenovirus expressing FKBP5 (Ad-FKBP5) or control adenovirus (Ad-Ctrl) one day after CTX injury. Muscles were harvested six days post-injection of adenovirus and subjected to western blot analysis with an anti-FKBP5 antibody. GAPDH was used as a loading control. **C.** Immunohistochemical staining for FKBP5 in muscles infected with Ad-Ctrl or Ad-FKBP5. Images are representative of 3 different muscles for each group. Black arrows indicate FKBP5 overexpressing cells. Scale bars: 20 μm.
